# High expression of SIGLEC7 may promote M2-type macrophage polarization leading to adverse prognosis in glioma patients

**DOI:** 10.3389/fimmu.2024.1411072

**Published:** 2024-08-15

**Authors:** Wenhao An, Changyuan Ren, Lei Yuan, Zhiqiang Qiu, Peishen Wang, Yanwen Cheng, Zi He, Xinye Han, Shouwei Li, Yihua An

**Affiliations:** ^1^ Department of Neurosurgery, Sanbo Brain Hospital, Capital Medical University, Beijing, China; ^2^ Department of Molecular Neuropathology, Beijing Neurosurgical Institute, Capital Medical University, Beijing, China; ^3^ Key Laboratory of Brain Science Research & Transformation in Tropical Environment of Hainan Province, Hainan Medical University, Haikou, China; ^4^ Division of Hematology and Oncology, Department of Pediatrics, Penn State College of Medicine, Hershey, PA, United States; ^5^ Department of Research and Development, Beijing Yihua Biotechnology Co., Ltd, Beijing, China

**Keywords:** SIGLEC7, glioma, M2 macrophage, prognostic indicator, tumor immunity

## Abstract

**Introduction:**

Gliomas are the most common primary intracranial tumors, known for their high invasiveness and destructiveness. Sialic acid-binding immunoglobulin-like lectin 7 (SIGLEC7) is present in various immune cells, especially macrophages, and significantly affects immune homeostasis and cancer cell response. However, research on the role and prognostic impact of SIGLEC7 in glioma patients is currently limited.

**Methods:**

We utilized transcriptomic data from 702 glioma patients in The Cancer Genome Atlas (TCGA) and 693 glioma patients in the Chinese Glioma Genome Atlas (CGGA), along with clinical samples we collected, to comprehensively investigate the impact of SIGLEC7 on glioma expression patterns, biological functions, and prognostic value. We focused on its role in glioma-related immune responses and immune cell infiltration and analyzed its expression at the single-cell level. Finally, we validated the role of SIGLEC7 in gliomas through tissue and cell experiments.

**Results:**

SIGLEC7 expression was significantly increased in glioma patients with malignant characteristics. Survival analysis indicated that glioma patients with high SIGLEC7 expression had significantly lower survival rates. Gene function analysis revealed that SIGLEC7 is primarily involved in immune and inflammatory responses and is strongly negatively correlated with tumor-associated immune regulation. Additionally, the expression of most immune checkpoints was positively correlated with SIGLEC7, and immune cell infiltration analysis clearly demonstrated a significant positive correlation between SIGLEC7 expression and M2 macrophage infiltration levels. Single-cell analysis, along with tissue and cell experiments, confirmed that SIGLEC7 enhances macrophage polarization towards the M2 phenotype, thereby promoting glioma invasiveness through the immunosuppressive effects of M2 macrophages. Cox regression analysis and the establishment of survival prediction models indicated that high SIGLEC7 expression is an unfavorable prognostic factor for glioma patients.

**Discussion:**

High SIGLEC7 expression predicts poor prognosis in glioma patients and is closely associated with M2 macrophages in the tumor environment. In the future, SIGLEC7 may become a promising target for glioma immunotherapy.

## 1 Introduction

Glioma, recognized for its high incidence and mortality rates, stands as the most prevalent primary intracranial tumor, constituting roughly 81% of malignant brain tumors (1, 2). Among them, the median overall survival time for WHO Grade III and Grade IV glioblastomas is 3 years and 15 months, respectively (3). They are characterized by rapid cell proliferation and angiogenesis, highly invasive behavior. Among various grades, the most malignant type, WHO Grade IV glioblastoma, alone represents 45% of all glioma cases (1, 4). Over the past few decades, glioma treatment has advanced to personalized approaches, including safe surgical resection guided by neuronavigation systems, postoperative radiotherapy, and molecular pathology-guided chemotherapy (5). Although treatment strategies continue to advance, patients with gliomas still face the challenges of recurrence and malignant progression, leading to poor prognosis (6). Moreover, the gradual development of chemoresistance by the tumor makes the prognosis bleak (7). Hence, there is an urgent need to continue exploring new approaches to glioma therapy. In recent years, immunotherapy, targeting tumor immune evasion mechanisms, has emerged as a highly promising research direction, bringing new hope for glioma treatment (8).

SIGLECs are a receptor family expressed in most immune cells, which recognize glycan moieties containing sialic acid on cell membranes and play crucial roles in regulating the activity and function of immune cells (9). According to previous studies, SIGLECs, by recognizing sialylated glycans expressed on glycoproteins in mammalian cells, can act as sentinels of self-immunity, thereby inhibiting innate and adaptive immune responses against self (10). Additionally, most SIGLECs contain immunoinhibitory motifs similar to the immune checkpoint receptor PD-1 and may contribute to dampening immune responses against tumors (9, 11). Researchers have confirmed that SIGLEC7 drives tumor-associated macrophages to promote tumor development in pancreatic cancer (12). In the latest research, SIGLEC9 has also been demonstrated to have potent therapeutic potential in glioblastoma (13). Speculating on the basis of the abundant presence of SIGLEC ligands on tumor cell surfaces, coupled with the inhibitory characteristics demonstrated by numerous SIGLECs (14), we propose SIGLEC7 as a potentially promising immunotherapeutic target for glioma.

Currently, there is no independent study based on a large number of clinical samples regarding the expression of SIGLEC7 in glioma patients. Therefore, we comprehensively explored the impact of SIGLEC7 on the expression patterns, biological functions, and prognostic value in glioma patients using the TCGA dataset of 702 cases and the CGGA dataset of 693 cases. We focused on its role in glioma-associated immune responses and immune cell infiltration, and analyzed its expression at the single-cell level. Finally, we validated our findings through tissue and cell experiments, aiming to provide strong theoretical evidence for current and future targeted SIGLEC7 therapies for glioma patients.

## 2 Result

### 2.1 The high expression of SIGLEC7 is correlated with the malignancy of gliomas

**Figure 1:**
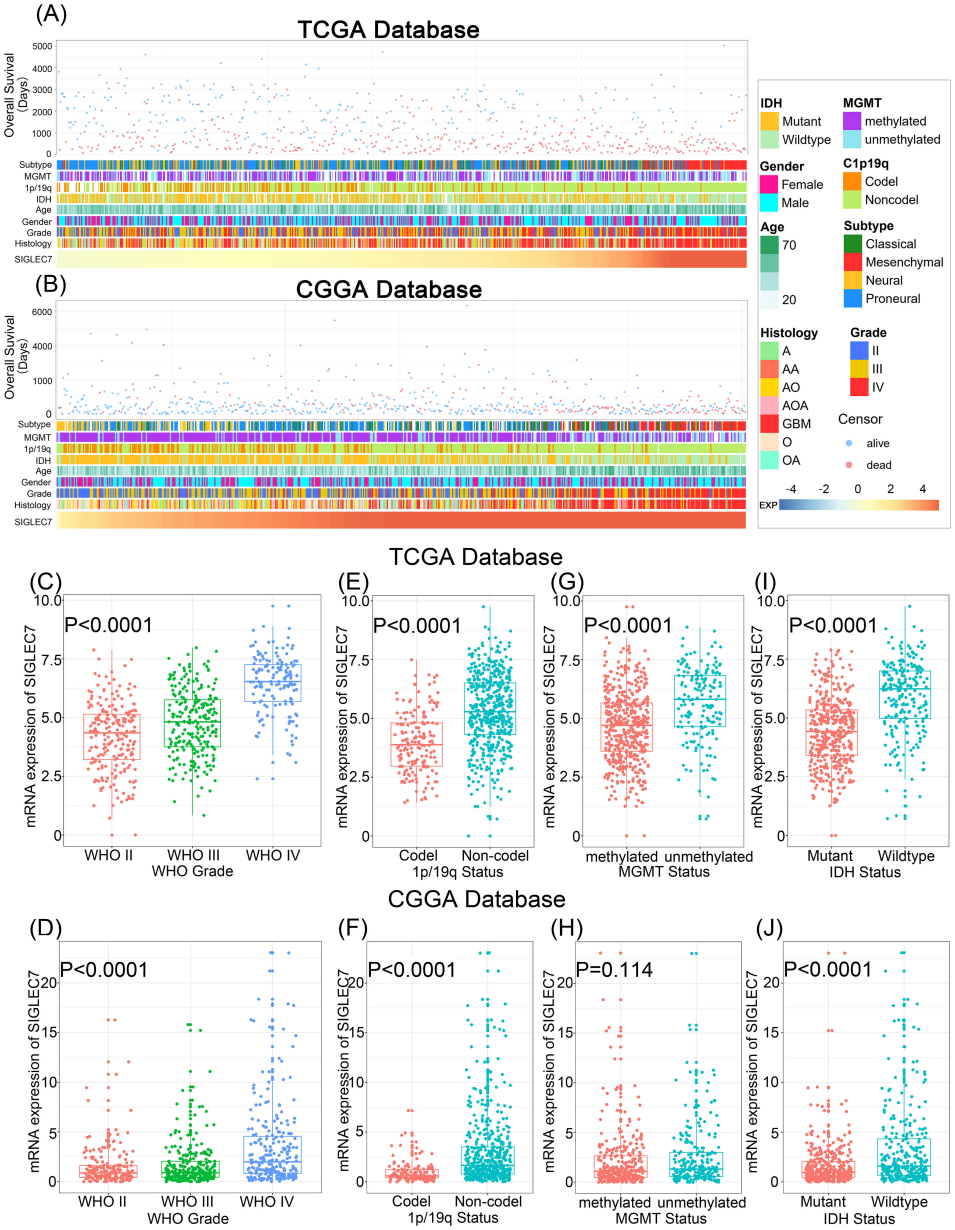
Correlation of SIGLEC7 with Clinicopathological Features of Gliomas. **(A)** Overview of the correlation between SIGLEC7 and clinicopathological features of gliomas in the TCGA database. **(B)** Overview of the correlation between SIGLEC7 and clinicopathological features of gliomas in the CGGA database. **(C, D)** Expression levels of SIGLEC7 gradually increase with glioma grade escalation in both TCGA and CGGA databases. Significance of differences was assessed using one-way ANOVA. **(E, F)** Gliomas without 1p/19q chromosomal co-deletion express higher levels of SIGLEC7 in both TCGA and CGGA databases. Significance of differences was assessed using unpaired t-tests. **(G, H)** Gliomas with O6-methylguanine-DNA methyltransferase (MGMT) promoter non-methylation express higher levels of SIGLEC7 in both TCGA and CGGA databases. Significance of differences was assessed using unpaired t-tests. **(I, J)** Gliomas without Isocitrate Dehydrogenase (IDH) mutations express higher levels of SIGLEC7 in both TCGA and CGGA databases. Significance of differences was assessed using unpaired t-tests.

To explore the association of SIGLEC7 with cancer, we employed GEPIA (Gene Expression Profiling Interactive Analysis) to compare SIGLEC7 expression levels across various tumors and their respective normal tissues. Our findings revealed a consistent elevation in SIGLEC7 expression across multiple tumor types, notably in LGG and GBM tissues, indicating a potential oncogenic role of SIGLEC7 in gliomas (**Supplementary Figure 1**). Based on the TCGA and CGGA databases, we analyzed the relationship between the expression level of SIGLEC7 and clinicopathological features of glioma patients, including histological diagnosis, WHO grade, gender, age, IDH mutation status, 1p/19q co-deletion status, MGMT promoter methylation status, and pathological subtypes, and described the patients’ relevant survival outcomes (**Figures 1A, B**). Contrasting these different groupings in the two datasets revealed that these results were not randomly distributed; notably, SIGLEC7 expression was significantly upregulated in higher-grade gliomas (**Figures 1C, D**). In clinical practice, IDH mutation status, 1p/19q co-deletion status, and MGMT promoter methylation status are of significant importance in predicting prognosis, treatment response, and clinical management of gliomas (15). In samples without 1p/19q co-deletion (**Figures 1E, F**), without MGMT promoter methylation (**Figures 1G, H**), and in IDH wild-type glioma patients (**Figures 1I, J**), we also found that patients had higher SIGLEC7 levels compared to the control group. Although the expression differences in these groupings in the CGGA database were not as pronounced as in the TCGA database, the trends were consistent between the two. Furthermore, we analyzed the survival outcomes of patients with different levels of SIGLEC7 expression (**Figures 1A, B**). The data showed that with increasing levels of SIGLEC7 expression, patient overall survival decreased, which was also observed in the CGGA database. In summary, this reveals that high expression of SIGLEC7 is associated with higher malignancy and poorer prognosis in glioma patients.

### 2.2 SIGLEC7 as a potential marker for mesenchymal subtypes of gliomas

**Figure 2:**
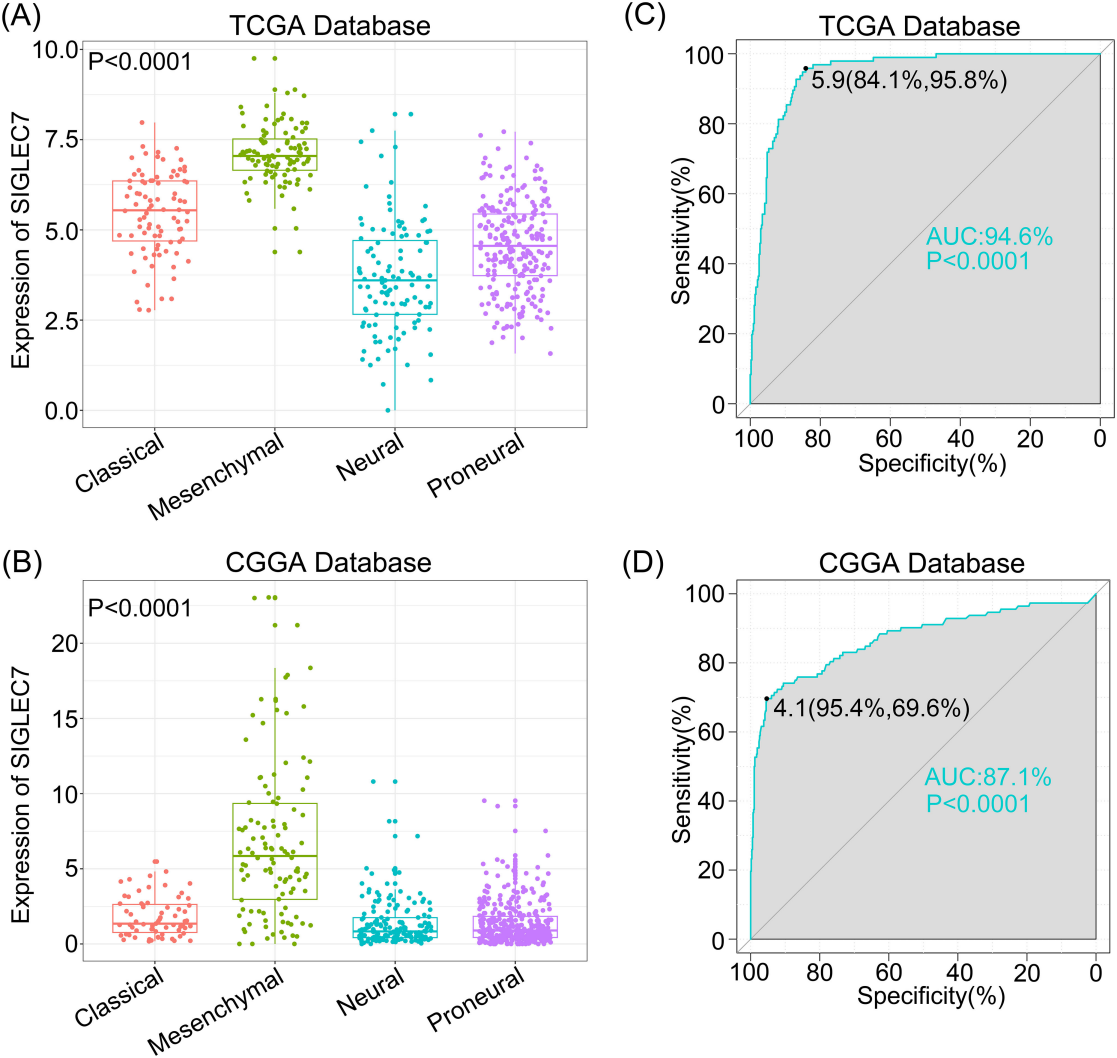
Specific Enrichment of SIGLEC7 in the mesenchymal subtype of Gliomas. **(A, C)** One-way ANOVA detects enrichment of SIGLEC7 in the mesenchymal subtype in the TCGA and CGGA databases. **(B, D)** Receiver Operating Characteristic (ROC) curves demonstrate the specificity of SIGLEC7 overexpression in the mesenchymal subtype of gliomas in the TCGA and CGGA databases, with the area under the curve (AUC) indicated.

Glioma transcriptional subtypes, analyzed based on transcriptomic data, provide more accurate molecular classifications and guide prognosis assessment and personalized treatment for gliomas, particularly the mesenchymal subtype, which suggests a malignant prognosis and has been widely recognized worldwide (16). Hence, we explored SIGLEC7 distribution across various subtypes utilizing the TCGA and CGGA databases. Our analysis revealed a notable upregulation of SIGLEC7 in the mesenchymal subtype compared to other subtypes (**Figures 2A, B**). To validate the specificity of our results, we utilized receiver operating characteristic (ROC) curves for evaluation (**Figures 2C, D**). As anticipated, the expression of SIGLEC7 in the mesenchymal subtype reached a significant area under the curve (AUC) value of 94.6% (P <0.0001) in the TCGA database, and 87.1% (P <0.0001) in the CGGA database. Therefore, we have reason to believe that the high expression of SIGLEC7 in the mesenchymal subtype of glioma could serve as one of the indicators for diagnosing the mesenchymal subtype of glioma. Additionally, these results align with our previous findings regarding the association of SIGLEC7 with the malignancy of glioma, further validating that high expression of SIGLEC7 predicts a poorer prognosis for patients.

### 2.3 SIGLEC7 influences immune function in glioma

**Figure 3:**
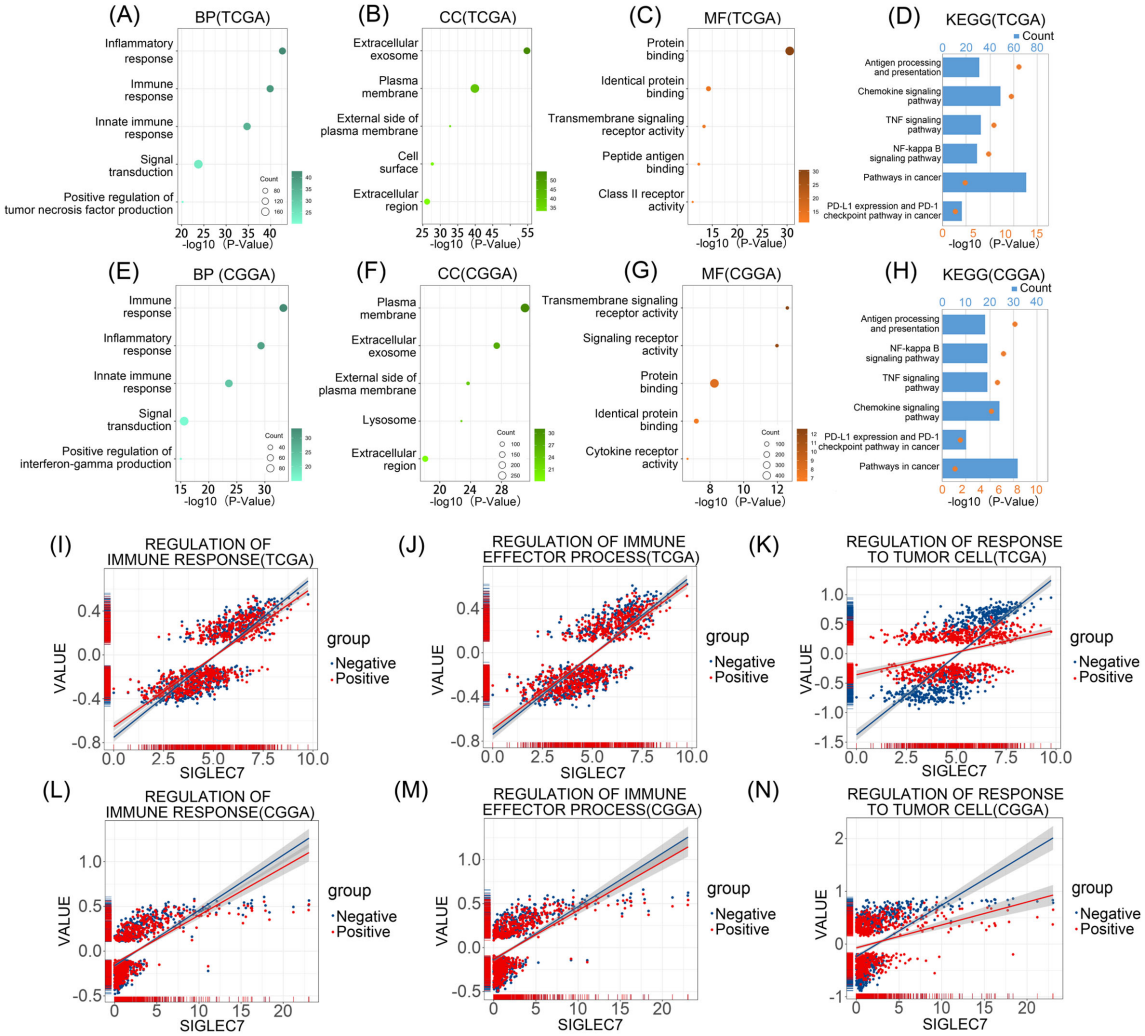
Enrichment Analysis Related to SIGLEC7 Gene. **(A-C, E-G)** Biological Processes (BP), Cellular Components (CC), and Molecular Functions (MF) most correlated with SIGLEC7 in TCGA and CGGA databases. **(D, H)** Kyoto Encyclopedia of Genes and Genomes (KEGG) pathway enrichment analysis related to SIGLEC7 in TCGA and CGGA databases. **(I-N)** Positive and negative regulation networks of immune processes associated with SIGLEC7 expression in TCGA and CGGA databases.

To explore the association between SIGLEC7 expression and biological functions in glioma, we conducted Pearson correlation analysis using TCGA and CGGA databases, identifying genes most positively correlated with SIGLEC7 expression (Pearson R > 0.5, p < 0.0001). In TCGA, 1464 genes were selected, and in CGGA, 687 genes were selected (**Supplementary Tables 1** and **2**). Subsequently, we performed gene ontology and KEGG pathway analyses based on these gene sets. In TCGA, biological processes ranked by significance from high to low were inflammation response, immune response, innate immune response, signal transduction, and positive regulation of tumor necrosis factor production (**Figure 3A**). The most correlated cellular component with SIGLEC7 was extracellular exosome (**Figure 3B**), and the associated molecular function was protein binding (**Figure 3C**). Similar enrichment results were obtained in the CGGA database (**Figures 3E-G**). KEGG analysis in both TCGA and CGGA databases showed close associations between SIGLEC7 and immune system function as well as tumor progression (**Figures 3D, H**). Further analysis by intersecting positively correlated genes from both databases consistently indicated that SIGLEC7 primarily participates in the immune and inflammatory processes of glioma (**Supplementary Figure 2**). Based on these findings, we speculate that SIGLEC7 predominantly engages in the immune response processes in glioma, consequently influencing its malignant progression.

### 2.4 SIGLEC7 is closely associated with negative regulation of response to tumor cells and expression of inhibitory immune checkpoints

**Figure 4:**
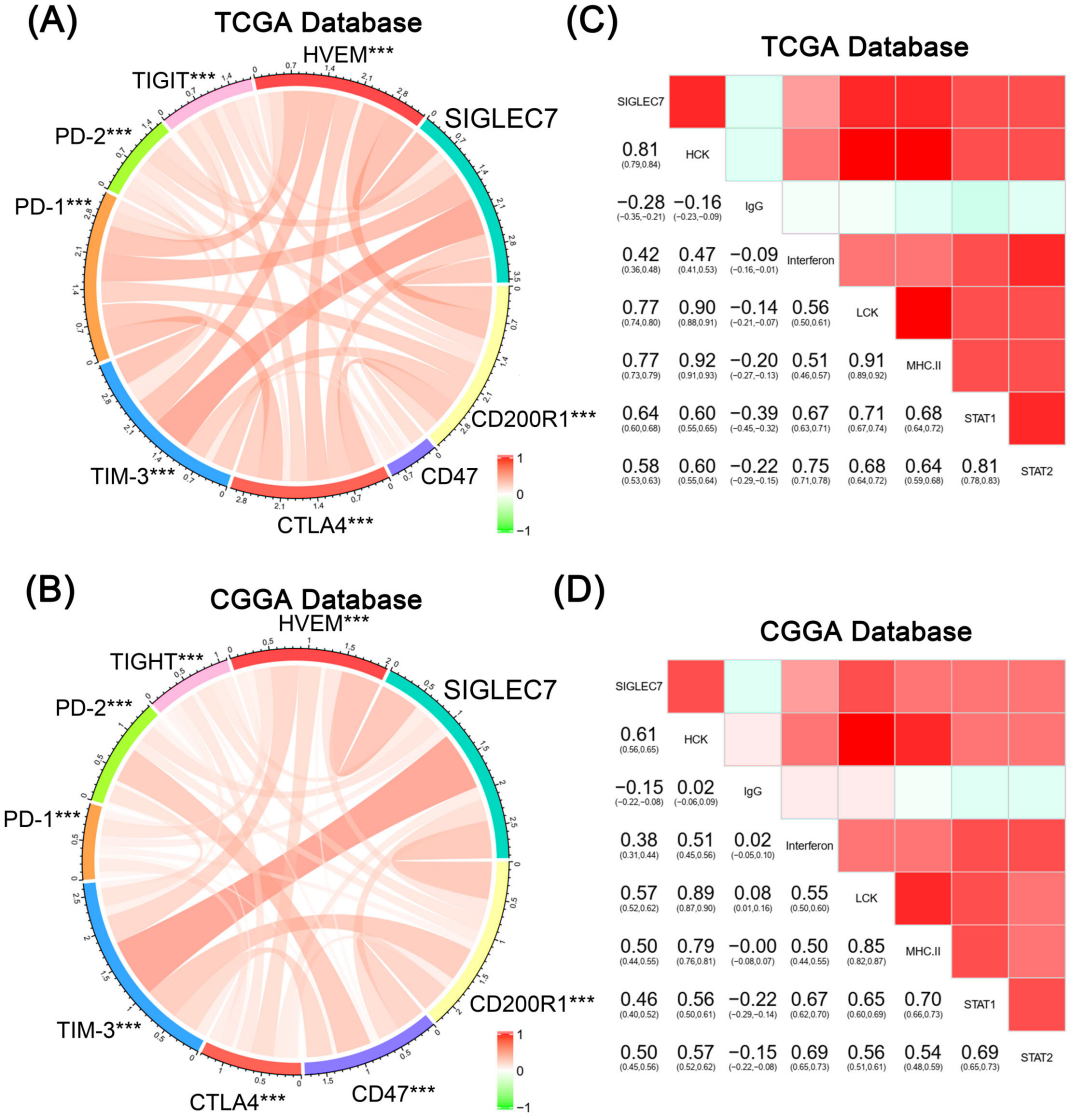
Relationship between SIGLEC7 and immune checkpoints and inflammatory responses. **(A, B)** Pearson correlation analysis between SIGLEC7 and immune checkpoints in TCGA and CGGA databases. The color bands represent the correlation, *** represents P<0.001, and unmarked indicates P>0.05. **(C, D)** Pearson correlation analysis matrix between SIGLEC7 and inflammation-related proteins in TCGA and CGGA databases. The depth of color in the lower left numerical values and upper right square represents the magnitude of the correlation coefficient.

To further validate our hypothesis, we conducted Gene Set Variation Analysis (GSVA) to compute the enrichment scores of gene sets in both TCGA and CGGA datasets. Specifically, we focused on the enrichment scores of three major categories: regulation of immune response, regulation of immune effector process, and regulation of responses to tumor cells (**Figures 3I-N**). Interestingly, we found that in the TCGA database, although the expression of SIGLEC7 was positively correlated with these enrichment scores, the correlation with inhibitory functions was often higher than that with promotive functions, particularly in the regulation of responses to tumor cells, where the correlation with negative modulation was more significant compared to positive regulation. Similar results were obtained in the CGGA database. Additionally, tumor cells often evade immunity by upregulating immune checkpoint genes (17). We investigated the relationship between SIGLEC7 and some well-known immune checkpoint genes using the TCGA and CGGA databases, including TIM-3, HVEM, CD200R1, CD47, TIGIT, CTLA4, PD-1, and PD-L2 (**Figures 4A, B**). The data revealed a significant positive correlation between these genes and SIGLEC7. Based on these results, we hypothesize that SIGLEC7 may collaborate with other immune checkpoints to collectively negatively regulate the organism’s responsiveness to tumors.

### 2.5 SIGLEC7 may promote the infiltration of M2 macrophages and be associated with inflammation dysregulation

**Figure 5:**
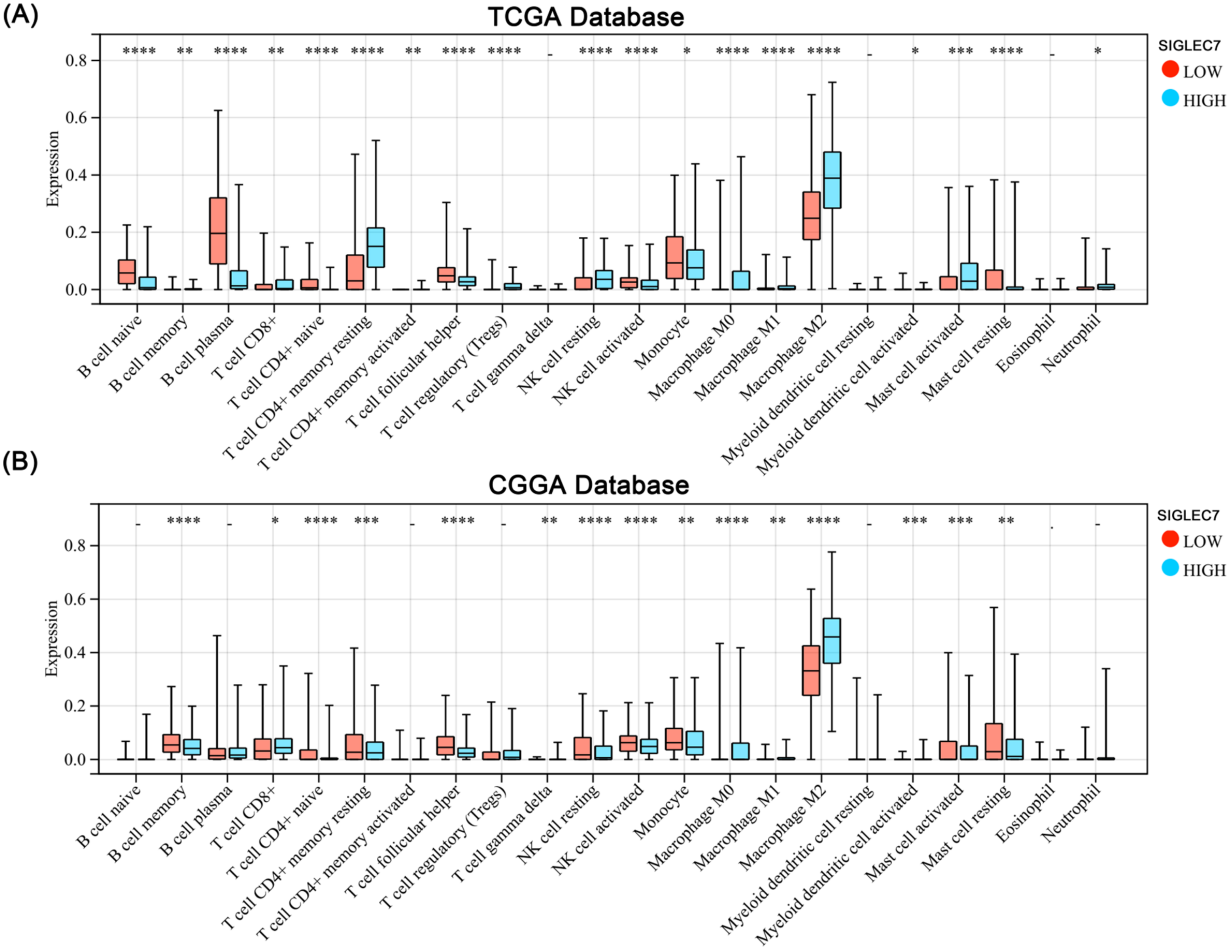
Impact of SIGLEC7 Expression on Immune Infiltration in Gliomas. **(A, B)** Comparison of levels of 22 immune cell infiltrations between glioma patients grouped by median SIGLEC7 expression in TCGA and CGGA databases. * represents p < 0.05, ** represents p < 0.01, *** represents p < 0.001, **** represents p < 0.0001.

To investigate the impact of SIGLEC7 on the inflammatory response in glioma, we conducted expression analyses of several immunoproteins of significance, including hematopoietic cell kinase (HCK), immunoglobulin G (IgG), interferon, lymphocyte-specific kinase (LCK), major histocompatibility complex (MHC), and signal transducer and activator of transcription 1/2 (STAT1/2) (18). According to the results (**Figures 4C, D**), SIGLEC7 exhibited a positive correlation with various inflammatory proteins associated with glioma, while showing a negative correlation with B cell IgG secretion. Additionally, to observe the impact of SIGLEC7 on immune cell infiltration in glioma, we imported data from two databases into the TIMER2.0 database for analysis and stratified patients into high and low expression groups based on the median expression of SIGLEC7. The results indicated a significant positive correlation between high SIGLEC7 expression groups and infiltration of M2-type macrophages in both databases (**Figures 5A, B**, **Supplementary Figure 3**). M2-type macrophages have been widely demonstrated to play crucial roles in suppressing immune responses and promoting tumor proliferation (19). Based on these findings, we conclude that upregulation of SIGLEC7 is significantly correlated with infiltration of M2-type macrophages, while also inhibiting certain functions of B cells. Therefore, we hypothesize that SIGLEC7 may exert immunosuppressive effects in glioma.

### 2.6 Single-cell analysis reveals predominant expression of SIGLEC7 in macrophages

**Figure 6:**
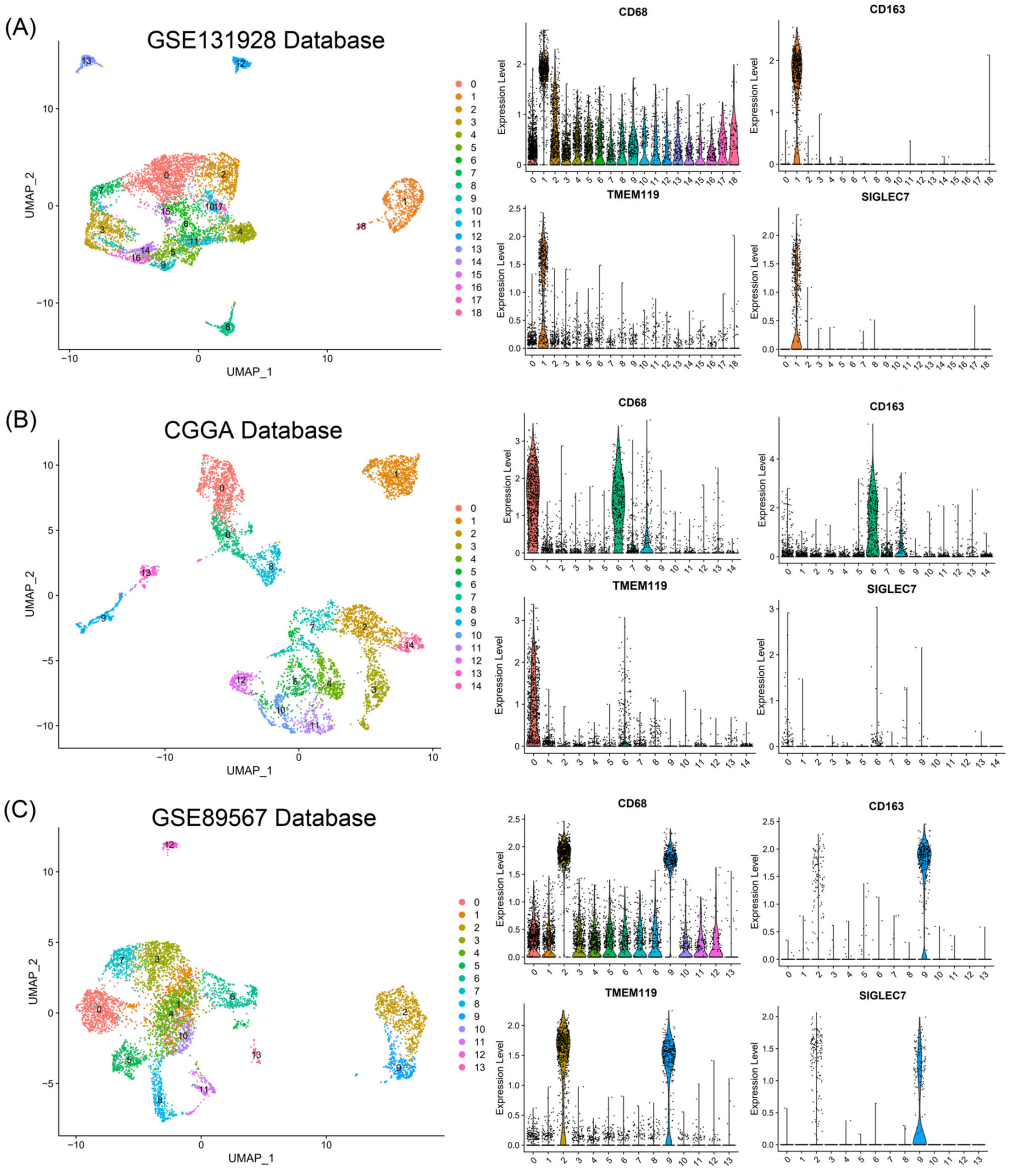
Relationship of SIGLEC7 with Macrophages and Microglia. **(A-C)** Relationship between SIGLEC7 and macrophages and microglia predicted based on typical markers CD68, CD163, and TMEM119 in GSE131928, CGGA, and GSE89567 databases.

Considering the significant positive correlation between SIGLEC7 and infiltration of M2 macrophages, we conducted single-cell sequencing analysis of different glioma patient types in the GSE131928, CGGA, and GSE89567 databases to explore their substantive connection. Initially, we performed simple clustering of glioma cells and conducted positional analysis using several typical markers. Among them, CD68, TMEM119, and CD163 are typical markers for macrophages, microglia, and M2 macrophages, respectively (20–22). During the analysis, we compared their distribution with that of SIGLEC7 and found a noticeable consistency between the distribution of SIGLEC7 and CD163, with this result being consistently observed across the three databases (**Figures 6A-C**). Additionally, using the Single Cell Portal database (GSE131928), we also observed predominant expression of SIGLEC7 in macrophages (**Supplementary Figure 4**). Based on this analysis and in conjunction with the preceding discussion, we hypothesize that the high expression of SIGLEC7 may lead to a predisposition of macrophages towards polarization into M2 macrophages, thereby altering the immune microenvironment. This alteration, through the immunosuppressive action of M2 macrophages, promotes the malignant progression of gliomas.

### 2.7 Cellular and histological experiments validate the role of SIGLEC7

**Figure 7:**
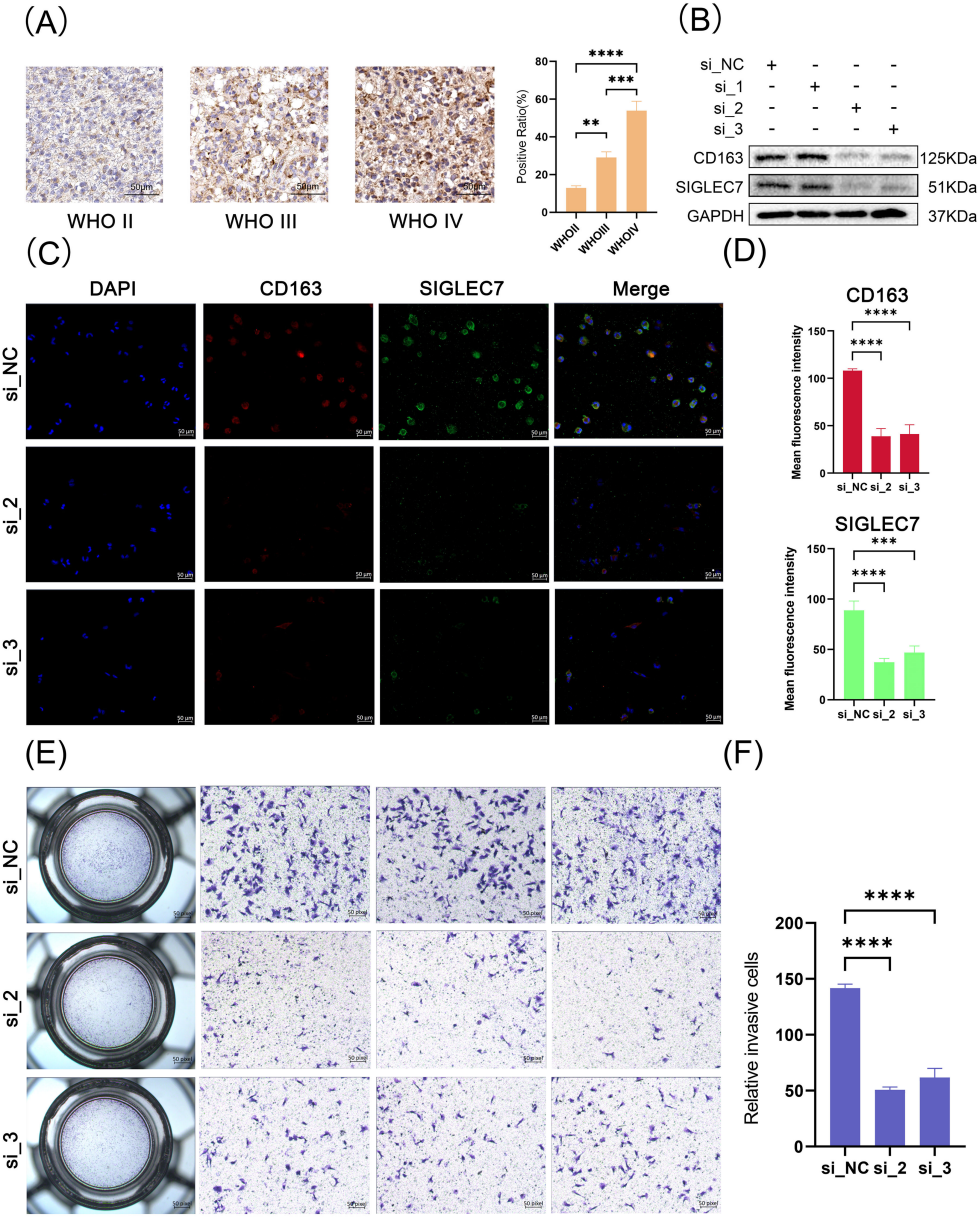
Tissue and Cellular Experiments Related to SIGLEC7. **(A)** Immunohistochemical staining of SIGLEC7 in different grades of glioma tissues, scale bar: 50 μm. **(B)** Western blot analysis of SIGLEC7 and CD163 expression in polarized macrophages after siRNA-mediated SIGLEC7 silencing. **(C)** Immunofluorescence staining of polarized macrophages. Blue fluorescence represents DAPI-stained cell nuclei, red fluorescence represents CD163 staining, and green fluorescence represents SIGLEC7 staining. The first row shows staining of normal M2 macrophages, while the second and third rows show staining after SIGLEC7 silencing. **(D)** Bar graph showing the average fluorescence intensity of CD163 and SIGLEC7-positive cells in macrophage immunofluorescence images. **(E, I)** Results of invasion assays after co-culture of macrophages with tumor cells. **p < 0.01; ***p < 0.001; ****p < 0.0001.

To confirm our previous speculations, we conducted relevant organizational and cellular experiments. Utilizing immunohistochemistry staining, we investigated the expression of SIGLEC7 in glioma tissues, with results consistent with RNA sequencing data, indicating an enriched expression of SIGLEC7 in higher-grade glioma tissues (**Figure 7A**). THP-1 cells are commonly used to simulate *in vivo* macrophages and tumor microenvironments (23). We successfully silenced the expression of SIGLEC7 and compared it with untreated macrophages. After polarization towards M2-type macrophages, the results showed reduced CD163 expression in the experimental group, seemingly validating our viewpoint on SIGLEC7 promoting M2-type macrophage polarization (**Figures 7B-D**). Subsequently, we co-cultured SIGLEC7-silenced polarized macrophages with glioma cells. Compared to the control group, the invasive ability of glioma cells in the experimental group decreased (**Figures 7E, F**).

### 2.8 Prognostic value of SIGLEC7 in gliomas

**Figure 8:**
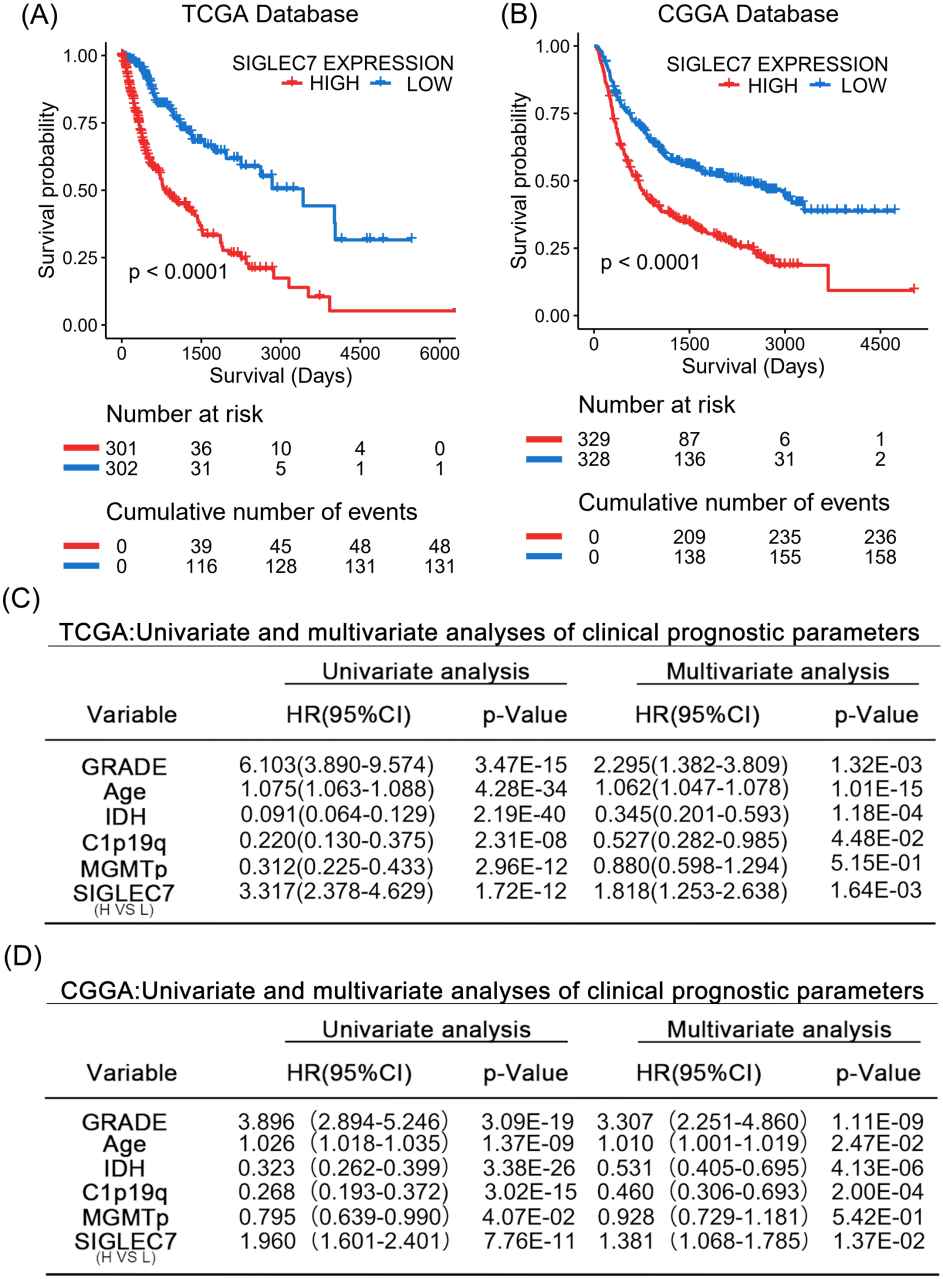
SIGLEC7 Marks Malignant Prognosis of Glioma Patients. **(A, B)** Impact of high and low expression of SIGLEC7 on the survival of glioma patients. Kaplan-Meier survival analysis was conducted on the TCGA and CGGA databases. **(C, D)** Univariate and multivariate analyses of parameters affecting clinical prognosis in the TCGA and CGGA databases.

**Figure 9:**
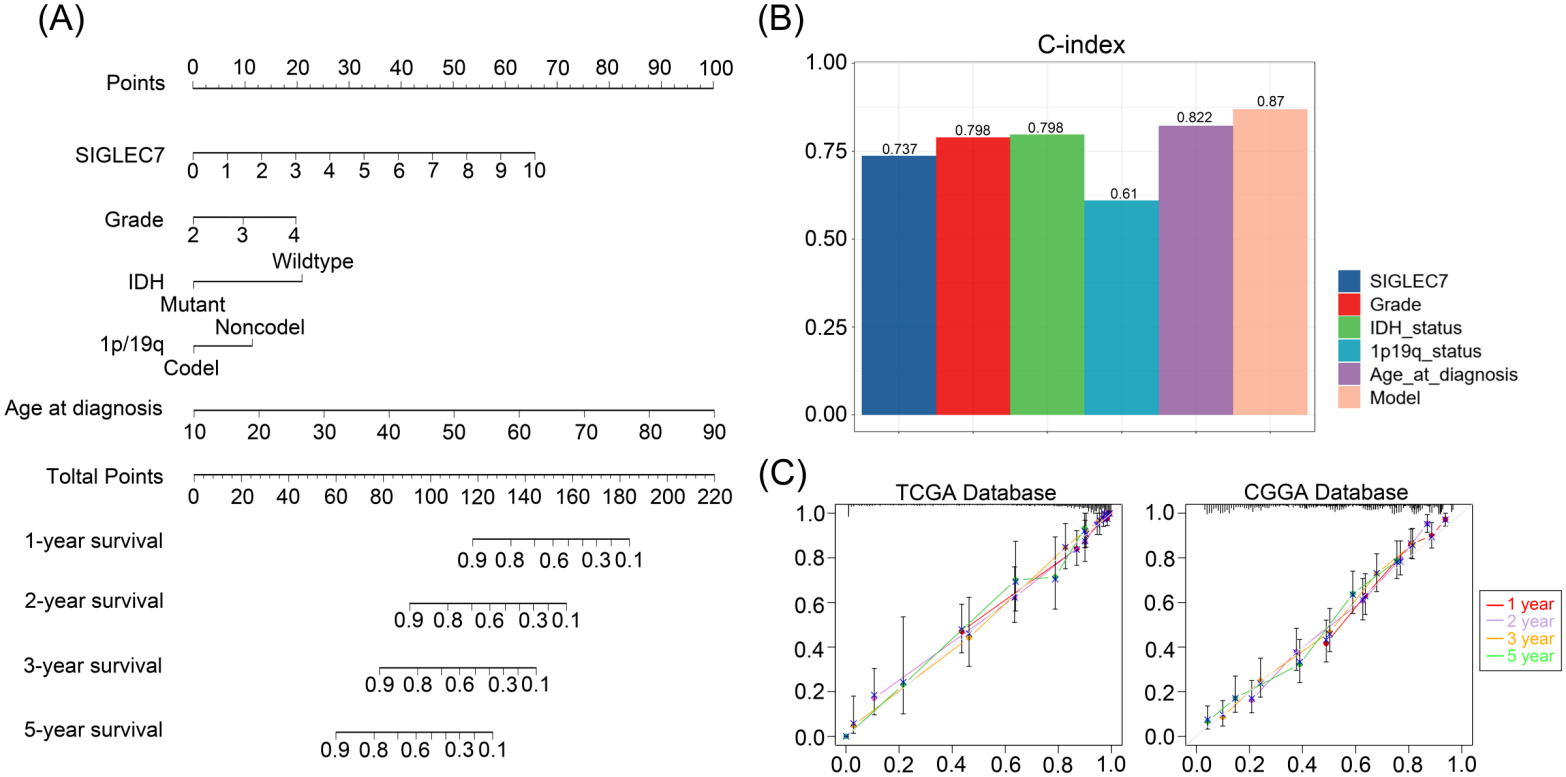
Personalized Prediction Model for Gliomas. **(A)** Nomogram predicting the 1-year, 2-year, 3-year, and 5-year survival probabilities of patients. **(B)** Evaluation of the model and predictive effects of individual indicators using the C-index. **(C)** Calibration plots showing the predicted probabilities of 1-year, 2-year, 3-year, and 5-year survival in TCGA and CGGA databases compared to actual outcomes.

Based on our research, we can confidently confirm a significant correlation between the expression of SIGLEC7 and poor prognosis in gliomas. Therefore, we further analyzed the prognostic predictive value of SIGLEC7 in glioma patients. We conducted analyses using Kaplan-Meier survival curves and Cox regression analysis on the TCGA and CGGA databases, yielding consistent results across both databases. The results revealed (**Figures 8A, B**) a clear distinction in survival curves when patients were grouped based on SIGLEC7 expression levels using the median cutoff. Patients with high SIGLEC7 expression exhibited markedly shorter overall survival, a trend corroborated across different grades of glioma patients (**Supplementary Figure 5**). Additionally, in Cox regression analysis, we included SIGLEC7 expression levels, WHO grade, age at diagnosis, IDH status, 1p/19q co-deletion status, and MGMT promoter methylation status as factors in both univariate and multivariate survival analyses, further confirming the significant prognostic impact of SIGLEC7 expression levels in glioma patients (**Figures 8C, D**). Finally, utilizing the TCGA database, we constructed a survival prediction model for glioma patients. This model incorporated SIGLEC7 expression levels, WHO grade, IDH mutation status, 1p/19q status, and patient age, demonstrating the probabilities of patient survival at 1, 2, 3, and 5 years under various conditions (**Figure 9A**). At the predictive level, the model’s Harrell’s C-index exceeded that of other factors within the model (**Figure 9B**), and further validation of the model’s predictive accuracy was achieved through calibration curves using data from both databases (**Figure 9C**).

## 3 Discussion

Glioma is one of the most aggressive and challenging tumors in humans (24). Despite numerous efforts to reduce mortality, it remains a daunting gap to bridge (5, 25). Over the past few decades, surgical resection of the tumor followed by radiotherapy and temozolomide chemotherapy has been the mainstay of glioma treatment (26). However, overall efficacy, particularly in glioma patients, has been unsatisfactory, with temozolomide, a first-line chemotherapy agent, demonstrating significant resistance (27–29). With the increasing attention and significant developments in precision medicine and targeted therapy (30), new hope has emerged. However, despite our comprehensive understanding of the molecular drivers of glioma, the effectiveness of single-targeted therapies remains limited (31). This is due to the close collaboration of tumor cells with the complex and diverse tumor microenvironment (TME), leading to this grim prognosis and making tumor-centric treatment approaches difficult to achieve (32). Among these, the glioma immune microenvironment (GIM), composed of glioma cells, stromal cells, and immune cells, plays a crucial role in glioma development and drug resistance (33, 34). The lack of significant progress in glioma treatment fundamentally lies in cancer cells’ adoption of the cleverest strategy—immune evasion (35). Therefore, focusing on the immune microenvironment of glioma, identifying immune checkpoint targets, and enhancing the effectiveness of immunotherapy are the focal points of future clinical treatment.

A wealth of evidence supports the involvement of SIGLEC7 in immune signaling, exerting inhibitory effects in regulating immune balance and serving as a checkpoint in cancer immune responses (36, 37). In recent years, SIGLEC7 has been found to be widely expressed in immune cells such as macrophages, dendritic cells, NK cells, mast cells, and eosinophils (38). As a ligand of SIGLEC7, total Sia, including sialylated glycolipids, gangliosides, and mucins (MUC), a highly sialylated glycoprotein family, has been demonstrated to be abundantly expressed in various types of tumor cells (39). Therefore, in the treatment of glioma patients, it is reasonable to consider the potential of SIGLEC7 in future treatment strategies.

In this study, we collected information from glioma patients in the TCGA database and the 693 cohort from the CGGA database, conducted comprehensive analysis after excluding patients with incomplete data. Based on patients’ mRNA-seq expression data, our findings indicate significant enrichment of SIGLEC7 expression in high-grade gliomas. Furthermore, among the four glioma subtypes, the mesenchymal subtype, which exhibits the highest malignancy, showed more pronounced enrichment. Additionally, SIGLEC7 was highly expressed in IDH wild-type glioma patients, those without 1p/19q co-deletion, and those without MGMT promoter methylation. To visually illustrate the relationship with patient survival time, we conducted survival analysis, revealing a significant decrease in survival time in the high-expression group. In summary, high expression of SIGLEC7 implies higher malignancy and poorer prognosis. Furthermore, we performed gene ontology and KEGG analyses on SIGLEC7-related gene sets to explore its most relevant biological functions. The primary biological processes were inflammation response and immune response. Interestingly, through our analysis, SIGLEC7 showed higher negative regulation correlation than positive regulation in the categories of regulation of immune response, regulation of immune effector process, and regulation of responses to tumor cells. In KEGG analysis, SIGLEC7 was mostly associated with tumor immune processes, although there were pathways promoting tumor expression. Previous studies have shown that tumor cells often evade the immune system by upregulating immune checkpoint genes (18). We investigated the relationship between SIGLEC7 and well-known immune checkpoints such as TIM-3, HVEM, CD200R1, CD47, TIGIT, CTLA4, and PD-1, revealing a significant positive correlation. We speculate that SIGLEC7 may promote glioma progression by upregulating other immune checkpoints and its immunosuppressive characteristics. Subsequently, we conducted a more detailed study on the inflammatory and immune response-related functions of SIGLEC7, selecting several representative clusters of immune protein expression genes for analysis. SIGLEC7 showed positive correlation with various immune functions but inhibited B cell IgG secretion. Furthermore, infiltration analysis of immune cells provided a more intuitive demonstration of the promotion of glioma development by SIGLEC7, showing a significant positive correlation between SIGLEC7 expression and M2 macrophage infiltration levels. M2 macrophages have been widely proven to play an important role in suppressing immune responses and promoting tumor proliferation (19). Combined with our single-cell sequencing analysis showing predominant expression of SIGLEC7 on macrophages, we speculate that SIGLEC7 promotes polarization of M2 macrophages, reshaping the microenvironment, and promoting glioma invasion through the immunosuppressive effect of M2 macrophages. Moreover, through tissue and cell experiments, we demonstrated the close correlation between SIGLEC7 and M2 macrophages. Finally, Cox regression analysis and establishment of survival prediction models proved that high expression of SIGLEC7 is indeed a significant adverse prognostic factor for glioma patients.

Overall, based on large-scale clinical samples, our study comprehensively explored the impact of SIGLEC7 on glioma expression patterns, biological functions, and prognostic value. We elucidated and analyzed its role in glioma-related immune responses, immune cell infiltration, and expression at the single-cell level. However, the level of this study is limited, and many questions remain unanswered. We only briefly analyzed the reasons for SIGLEC7 promoting tumor progression, and further exploration of deeper mechanisms is needed in the future.

Treatment strategies for glioma are increasingly moving towards combination therapy, integrating various treatment modalities such as surgical resection, radiotherapy, chemotherapy, immunotherapy, and targeted therapy (40). This study explores the close association between SIGLEC7 and the malignant development of glioma, providing a more solid theoretical basis for targeted immunotherapy against SIGLEC7. In the future, we look forward to the combination of targeted therapies targeting SIGLEC7 becoming an important strategy for glioma combination therapy, providing more effective treatment options for patients.

## 4 Materials and methods

### 4.1 Data acquisition

This study collected all selected cases of WHO grade II to IV gliomas from The Cancer Genome Atlas (TCGA) database (702 cases) (http://cancergenome.nih.gov/) and the Chinese Glioma Genome Atlas (CGGA) database (693 cases) (http://www.cgga.org.cn). All clinical information of patients was presented in **Table 1**. In the analysis process, cases with incomplete information were handled by treating them as missing data. Single-cell data were obtained from the CGGA database as well as the Gene Expression Omnibus (GEO) databases GSE89567 and GSE131928 (https://www.ncbi.nlm.nih.gov/geo/). Samples for immunohistochemistry were obtained from Sanbo Brain Hospital Capital Medical University with approval from the hospital’s board/ethics committee.

**Table 1:** Clinical information of patients.

Characteristics (TCGA)	No. of Patients (n=702)	Characteristics (CGGA)	No. of Patients (n=693)
Age		Age	
<45	284	<45	382
≥ 45	325	≥ 45	310
NA	93	NA	1
Gender		Gender	
Male	354	Male	398
Female	255	Female	295
NA	93	NA	0
WHO Grade		WHO Grade	
Grade II	216	Grade II	188
Grade III	241	Grade 111	255
Grade IV	152	Grade IV	249
NA	93	NA	1
Subtypes		Subtypes	
Proneural	238	Proneural	352
Neural	111	Neural	162
Classical	86	Classical	67
Mesenchymal	96	Mesenchymal	112
NA	171	NA	0
IDH mutation		IDH mutation	
Mutation	428	Mutation	356
Wildtype	234	Wildtype	286
NA	40	NA	51
1p/19q codeletion		1p/19q codeletion	
Codeletion	169	Codeletion	145
Non-codeletion	495	Non-codeletion	478
NA	38	NA	70
MGMT methylation		MGMT methylation	
Methylation	492	Methylation	315
Unmethyl lation	168	Unmethy lation	227
NA	42	NA	151

### 4.2 Enrichment analysis of relevant genes

Pearson correlation coefficient was utilized to select the gene list most closely related to SIGLEC7. Subsequently, this list was uploaded to https://david.ncifcrf.gov/ for Gene Ontology (GO) and Kyoto Encyclopedia of Genes and Genomes (KEGG) pathway analysis, with results visualized using heatmaps.

### 4.3 Gene set variation analysis

Biological process gene sets were downloaded from the GSEA website (https://www.gsea-msigdb.org/gsea). The enrichment scores for each biological process per patient were calculated using the default parameters of the R package GSVA. Interested biological processes were visualized using correlation scatter plots.

### 4.4 Immune cell infiltration analysis

The patient mRNA expression matrices were uploaded to the online analysis platform TIMER2.0 (http://timer.cistrome.org) for analysis. Utilizing the CIBERSORT algorithm, the abundance of 22 immune cell types infiltrating the tumors was obtained. Subsequently, these results were visualized using box plots and bar graphs.

### 4.5 Single-cell analysis of SIGLEC7

Three glioma patient databases were subjected to cell clustering analysis using the R package Seurat. Initially, poor-quality cells and genes were filtered out, followed by batch effect removal and data normalization. Dimensionality reduction of cells was achieved using Uniform Manifold Approximation and Projection (UMAP), with cluster results visualized in scatter plots.

### 4.6 Immunohistochemical staining

Glioma tissue sections, with a thickness of four micrometers, were first immersed in Tris-EDTA buffer at pH 9.0. Antigen retrieval was carried out by heating the sections in a water bath at 100°C for 15 minutes. Endogenous peroxidases were then blocked using a 3% hydrogen peroxide solution. Following this, goat serum was applied for 30 minutes to prevent nonspecific binding. The sections were then treated with the appropriate concentration of SIGLEC7 antibody and co-incubated overnight. Finally, secondary antibody staining, conjugated with HRP, was performed, and the results were observed using an optical microscope.

### 4.7 THP-1 cell culture and polarization induction

The THP-1 cells were cultured at 37°C in a 5% CO2 atmosphere in RPMI1640 medium supplemented with 10% fetal bovine serum, 1% penicillin-streptomycin, and 0.1 mM β-mercaptoethanol. To induce differentiation into M0 macrophages, cells were treated with 100 ng/mL phorbol myristate acetate (PMA) for 48 hours. Subsequently, polarization into M2 macrophages was achieved by co-incubation with 20 ng/ml IL-4 and 20 ng/ml IL-13 in the presence of PMA. Concurrently, siRNA-NC and siRNA-SIGLEC7 were added to the control and experimental groups, followed by a 48-hour co-incubation.

### 4.8 Western blotting

After polarization and siRNA transfection, total protein was extracted from THP-1 cells. The proteins were quantified using the BCA method and then denatured by boiling with 5x loading buffer. Following this, protein samples were subjected to electrophoresis for separation and transferred onto membranes. These membranes were then blocked with 5% skim milk at room temperature for 2 hours. Subsequently, they were incubated overnight at 4°C with primary antibodies against SIGLEC7 (immunoway, YT5281, 1:1000), CD163 (Abcam, ab156769, 1:1000), and GAPDH (immunoway, YM3029, 1:5000). After washing with TBST buffer, the membranes were incubated with appropriate secondary antibodies, and chemiluminescence was detected using the Bio-Rad imaging system.

### 4.9 Immunofluorescence assay

Cells were fixed with 4% paraformaldehyde for 20 minutes after multiple washes with PBS, permeabilized with 1% Triton X-100 for 10 minutes, and then blocked with goat serum for 20 minutes. Primary antibody solution containing SIGLEC7 (immunoway, YT5281, 1:200) and CD163 (Abcam, ab156769, 1:200) was added and incubated overnight at 4°C. Subsequently, cells were incubated with secondary antibodies (Goat anti-Mouse IgG (H+L) Cross-Adsorbed Secondary Antibody, Alexa Fluor™ 488, A11001, 1:200; Goat anti-Mouse IgG (H+L) Cross-Adsorbed Secondary Antibody, Alexa Fluor™ 594, A32740, 1:200) for 1 hour at room temperature. After three PBS washes, cells were mounted with Prolong™ Diamond Antifade Mountant containing DAPI (Invitrogen, P36962), and images were captured using confocal microscopy.

### 4.10 Invasion assay

Transwell invasion assays were performed using 8μm polycarbonate membrane inserts pre-coated with Matrigel (Corning) in a 24-well plate. LN229 cells (1×10^5 cells/100μl) were seeded in the upper chamber, while M2 cells (1×10^5 cells/500μl) were added to the lower chamber containing 2% FBS medium. After 10 hours, non-invading cells were removed, and the invaded cells were fixed with 4% PFA and stained with 1% crystal violet. Images were captured using a microscope after cleaning the inserts.

### 4.11 Statistical analysis and visualization

This study primarily utilized R software (version 4.2.3, Windows platform) and IBM SPSS Statistics (version 27.0.1, Windows platform) for statistical analysis and chart generation. Various R packages were employed including ggplot2, pheatmap, circlize, GSVA, ggpubr, survminer, survival, corrgram, dplyr, Seurat, patchwork, and pROC for data visualization and analysis. Additionally, IBM SPSS Statistics was utilized for both univariate and multivariate Cox regression analysis. Pearson correlation analysis was conducted to assess the correlation between two sets of data. The significance of differences between two groups was evaluated using Student’s t-test, while one-way analysis of variance (ANOVA) was employed to assess differences among more than two groups. A p-value < 0.05 was considered statistically significant.

## Data Availability

The datasets presented in this study can be found in online repositories. The names of the repository/repositories and accession number(s) can be found in the article/**Supplementary Material**.

## References

[1] Ostrom QT Bauchet L Davis FG Deltour I Fisher JL Langer CE. The epidemiology of glioma in adults: a “state of the science” review. Neuro Oncol. (2014) 16:896–913. doi: 10.1093/neuonc/nou087 24842956 PMC4057143

[2] Ghouzlani A Kandoussi S Tall M Reddy KP Rafii S Badou A. Immune checkpoint inhibitors in human glioma microenvironment. Front Immunol. (2021) 12:679425. doi: 10.3389/fimmu.2021.679425 34305910 PMC8301219

[3] Gusyatiner O Hegi ME. Glioma epigenetics: From subclassification to novel treatment options. Semin Cancer Biol. (2018) 51:50–8. doi: 10.1016/j.semcancer.2017.11.010 29170066

[4] Peng Z Liu C Wu M. New insights into long noncoding RNAs and their roles in glioma. Mol Cancer. (2018) 17(1):61. doi: 10.1186/s12943-018-0812-2 29458374 PMC5817731

[5] Van Meir EG Hadjipanayis CG Norden AD Shu HK Wen PY Olson JJ. Exciting new advances in neuro-oncology: the avenue to a cure for Malignant glioma. CA Cancer J Clin. (2010) 60:166–93. doi: 10.3322/caac.20069 PMC2888474 20445000

[6] Xu X Li L Luo L Shu L Si X Chen Z. Opportunities and challenges of glioma organoids. Cell Commun Signal. (2021) 19:102. doi: 10.1186/s12964-021-00777-0 34635112 PMC8504127

[7] Yang Y Liu L Tian Y Gu M Wang Y Ashrafizadeh M. Autophagy-driven regulation of cisplatin response in human cancers: Exploring molecular and cell death dynamics. Cancer Letters. (2024) 587:216659. doi: 10.1016/j.canlet.2024.216659 38367897

[8] Daubon T Hemadou A Romero Garmendia I Saleh M. Glioblastoma immune landscape and the potential of new immunotherapies. Front Immunol. (2020) 11:585616. doi: 10.3389/fimmu.2020.585616 33154756 PMC7591769

[9] van Houtum EJH Büll C Cornelissen LAM Adema GJ. Siglec signaling in the tumor microenvironment. Front Immunol. (2021) 12:790317. doi: 10.3389/fimmu.2021.790317 34966391 PMC8710542

[10] Paulson JC Macauley MS Kawasaki N. Siglecs as sensors of self in innate and adaptive immune responses. Ann N Y Acad Sci. (2012) 1253:37–48. doi: 10.1111/j.1749-6632.2011.06362.x 22288608 PMC3335958

[11] Fraschilla I Pillai S. Viewing Siglecs through the lens of tumor immunology. Immunol Rev. (2017) 276:178–91. doi: 10.1111/imr.12526 PMC5860639 28258691

[12] Rodriguez E Boelaars K Brown K Eveline Li RJ Kruijssen L Bruijns SCM. Sialic acids in pancreatic cancer cells drive tumourassociated macrophage differentiation via the Siglec receptors Siglec-7 and Siglec-9. Nat Commun. (2021) 12(1):1270. doi: 10.1038/s41467-021-21550-4 33627655 PMC7904912

[13] Schmassmann P Roux J Buck A Tatari N Hogan S Wang J. Targeting the Siglec-sialic acid axis promotes antitumor immune responses in preclinical models of glioblastoma. Sci Transl Med. (2023) 15:eadf5302. doi: 10.1126/scitranslmed.adf5302 37467314

[14] Santegoets KCM Gielen PR Büll C Schulte BM Kers-Rebel ED Küsters B. Expression profiling of immune inhibitory Siglecs and their ligands in patients with glioma. Cancer Immunol Immunother. (2019) 68:937–49. doi: 10.1007/s00262-019-02332-w PMC6529385 30953118

[15] Wiestler B Capper D Hovestadt V Sill M Jones DTW Hartmann C. Assessing CpG island methylator phenotype, 1p/19q codeletion, and MGMT promoter methylation from epigenome-wide data in the biomarker cohort of the NOA-04 trial. Neuro-Oncology. (2014) 16:1630–8. doi: 10.1093/neuonc/nou138 PMC4232086 25028501

[16] Verhaak RGW Hoadley KA Purdom E Wang V Qi Y Wilkerson MD. Integrated genomic analysis identifies clinically relevant subtypes of glioblastoma characterized by abnormalities in PDGFRA, IDH1, EGFR, and NF1. Cancer Cell. (2010) 17:98–110. doi: 10.1016/j.ccr.2009.12.020 20129251 PMC2818769

[17] Nirschl CJ Drake CG. Molecular pathways: coexpression of immune checkpoint molecules: signaling pathways and implications for cancer immunotherapy. Clin Cancer Res. (2013) 19:4917–24. doi: 10.1158/1078-0432.CCR-12-1972 PMC4005613 23868869

[18] Di W Fan W Wu F Shi Z Wang Z Yu M. Clinical characterization and immunosuppressive regulation of CD161 (KLRB1) in glioma through 916 samples. Cancer Sci. (2022) 113:756–69. doi: 10.1111/cas.15236 PMC8819299 34881489

[19] Zhou W Ke SQ Huang Z Flavahan W Fang X Paul J. Periostin secreted by glioblastoma stem cells recruits M2 tumour-associated macrophages and promotes Malignant growth. Nat Cell Biol. (2015) 17:170–82. doi: 10.1038/ncb3090 PMC4312504 25580734

[20] Mercurio D Fumagalli S Schafer MKH Pedragosa J Ngassam LDC Wilhelmi V. Protein expression of the microglial marker tmem119 decreases in association with morphological changes and location in a mouse model of traumatic brain injury. Front Cell Neurosci. (2022) 16:820127. doi: 10.3389/fncel.2022.820127 35221925 PMC8866855

[21] Yamaguchi Y Gibson J Ou K Lopez LS Ng RH Leggett N. PD-L1 blockade restores CAR T cell activity through IFN-γ-regulation of CD163+ M2 macrophages. J Immunother Cancer. (2022) 10:e004400. doi: 10.1136/jitc-2021-004400 35738799 PMC9226933

[22] Wang YY Jiang H Pan J Huang XR Wang YC Huang HF. Macrophage-to-myofibroblast transition contributes to interstitial fibrosis in chronic renal allograft injury. J Am Soc Nephrol. (2017) 28:2053–67. doi: 10.1681/ASN.2016050573 PMC5491278 28209809

[23] Chanput W Mes JJ Wichers HJ. THP-1 cell line: an in *vitro* cell model for immune modulation approach. Int Immunopharmacol. (2014) 23:37–45. doi: 10.1016/j.intimp.2014.08.002 25130606

[24] Touat M Idbaih A Sanson M Ligon KL. Glioblastoma targeted therapy: updated approaches from recent biological insights. Ann Oncol. (2017) 28:1457–72. doi: 10.1093/annonc/mdx106 PMC5834086 28863449

[25] Hu H Mu Q Bao Z Chen Y Liu Y Chen J. Mutational landscape of secondary glioblastoma guides MET-targeted trial in brain tumor. Cell. (2018) 175:1665–78.e18. doi: 10.1016/j.cell.2018.09.038 30343896

[26] Nicholson JG Fine HA. Diffuse glioma heterogeneity and its therapeutic implications. Cancer Discovery. (2021) 11:575–90. doi: 10.1158/2159-8290.CD-20-1474 33558264

[27] Bagley SJ Desai AS Linette GP June CH O’Rourke DM. CAR T-cell therapy for glioblastoma: recent clinical advances and future challenges. Neuro Oncol. (2018) 20:1429–38. doi: 10.1093/neuonc/noy032 PMC6176794 29509936

[28] van den Bent MJ Tesileanu CMS Wick W Sanson M Brandes AA Clement PM. Adjuvant and concurrent temozolomide for 1p/19q non-co-deleted anaplastic glioma (CATNON; EORTC study 26053-22054): second interim analysis of a randomised, open-label, phase 3 study. Lancet Oncol. (2021) 22:813–23. doi: 10.1016/S1470-2045(21)00090-5 PMC8191233 34000245

[29] Xue W Yang L Chen C Ashrafizadeh M Tian Y Sun R. Wnt/β-catenin-driven EMT regulation in human cancers. Cell Mol Life Sci. (2024) 81(1):79. doi: 10.1007/s00018-023-05099-7 38334836 PMC10857981

[30] Prados MD Byron SA Tran NL Phillips JJ Molinaro AM Ligon KL. Toward precision medicine in glioblastoma: the promise and the challenges. Neuro Oncol. (2015) 17:1051–63. doi: 10.1093/neuonc/nov031 PMC4490873 25934816

[31] Olson JJ Nayak L Ormond DR Wen PY Kalkanis SN Ryken TC. The role of targeted therapies in the management of progressive glioblastoma : a systematic review and evidence-based clinical practice guideline. J Neurooncol. (2014) 118:557–99. doi: 10.1007/s11060-013-1339-4 24740195

[32] Boussiotis VA Charest A. Immunotherapies for Malignant glioma. Oncogene. (2018) 37:1121–41. doi: 10.1038/s41388-017-0024-z PMC5828703 29242608

[33] Barthel L Hadamitzky M Dammann P Schedlowski M Sure U Thakur BK. Glioma: molecular signature and crossroads with tumor microenvironment. Cancer Metastasis Rev. (2022) 41:53–75. doi: 10.1007/s10555-021-09997-9 34687436 PMC8924130

[34] Zhou F Shi Q Fan X Yu R Wu Z Wang B. Diverse macrophages constituted the glioma microenvironment and influenced by PTEN status. Front Immunol. (2022) 13:841404. doi: 10.3389/fimmu.2022.841404 35265085 PMC8899089

[35] Lu Q Kou D Lou S Ashrafizadeh M Aref AR Canadas I. Nanoparticles in tumor microenvironment remodeling and cancer immunotherapy. J Hematol Oncol. (2024) 17:16. doi: 10.1186/s13045-024-01535-8 38566199 PMC10986145

[36] Gianchecchi E Arena A Fierabracci A. Sialic acid-siglec axis in human immune regulation, involvement in autoimmunity and cancer and potential therapeutic treatments. Int J Mol Sci. (2021) 22:5774. doi: 10.3390/ijms22115774 34071314 PMC8198044

[37] Hudak JE Canham SM Bertozzi CR. Glycocalyx engineering reveals a siglec-based mechanism for NK cell immunoevasion. Nat Chem Biol. (2014) 10:69–75. doi: 10.1038/nchembio.1388 24292068 PMC3893890

[38] Lock K Zhang J Lu J Lee SH Crocker PR. Expression of CD33-related siglecs on human mononuclear phagocytes, monocyte-derived dendritic cells and plasmacytoid dendritic cells. Immunobiology. (2004) 209:199–207. doi: 10.1016/j.imbio.2004.04.007 15481154

[39] Zheng Y Ma X Su D Zhang Y Yu L Jiang F. The roles of siglec7 and siglec9 on natural killer cells in virus infection and tumour progression. J Immunol Res. (2020) 2020:6243819. doi: 10.1155/2020/6243819 32322597 PMC7165337

[40] Yang K Wu Z Zhang H Zhang N Wu W Wang Z. Glioma targeted therapy: insight into future of molecular approaches. Mol Cancer. (2022) 21:39. doi: 10.1186/s12943-022-01513-z 35135556 PMC8822752

