# Supplementary material for: High expression of SIGLEC7 may promote M2-type macrophage polarization leading to adverse prognosis in glioma patients
Source: Front Immunol. 2024 Aug 15;15:1411072. doi: 10.3389/fimmu.2024.1411072 (PMC11357930; doi:10.3389/fimmu.2024.1411072)
Supplement: Supplementary file 1 [file DataSheet1.docx]

Supplementary Material

## Supplementary Figures


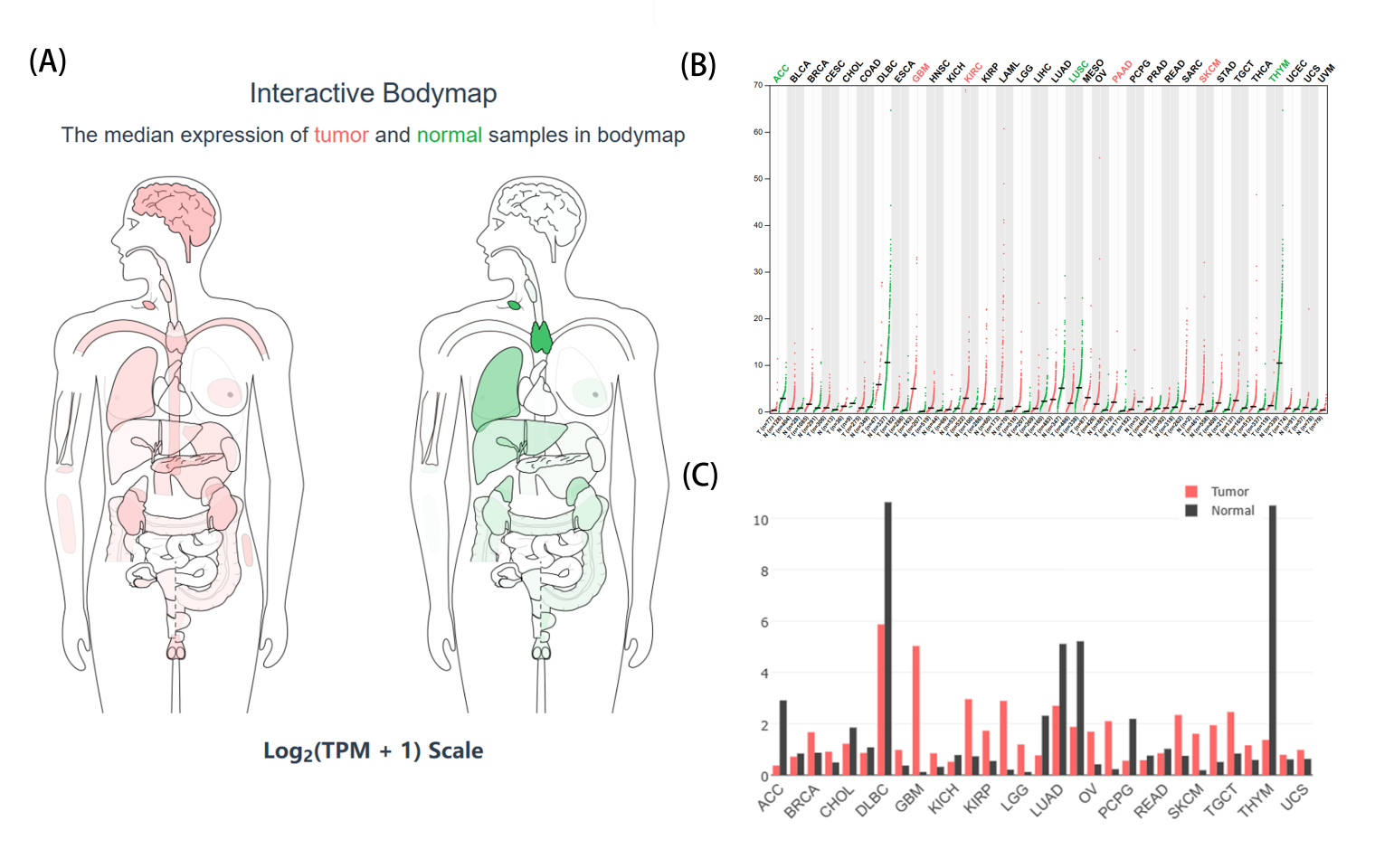


**Supplementary Figure 1**. High expression of SIGLEC7 in low-grade gliomas and glioblastomas. A, Expression distribution of SIGLEC7 in human tumor tissues and normal tissues. B and C: Expression of SIGLEC7 in various tumors and their corresponding normal tissues.


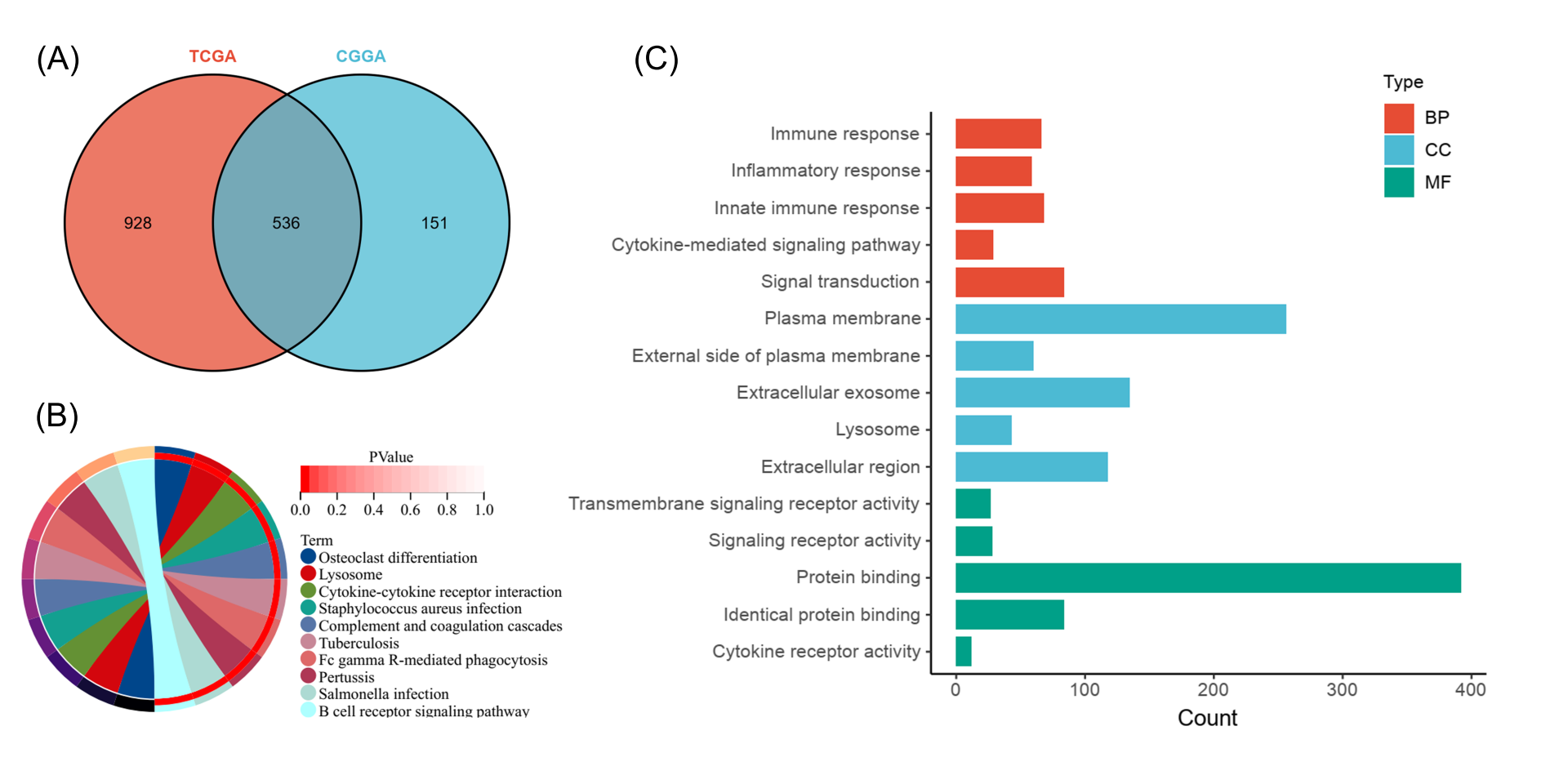


**Supplementary Figure 2**. Enrichment analysis of the intersection of SIGLEC7-associated genes in TCGA and CGGA databases. A, Venn diagram of the intersection of SIGLEC7-associated genes in TCGA and CGGA databases. B, Top 10 enriched KEGG pathways for intersection genes with the most significant P-values. C, Top 5 enriched biological processes (BP), cellular components (CC), and molecular functions (MF) for intersection genes with the most significant P-values.


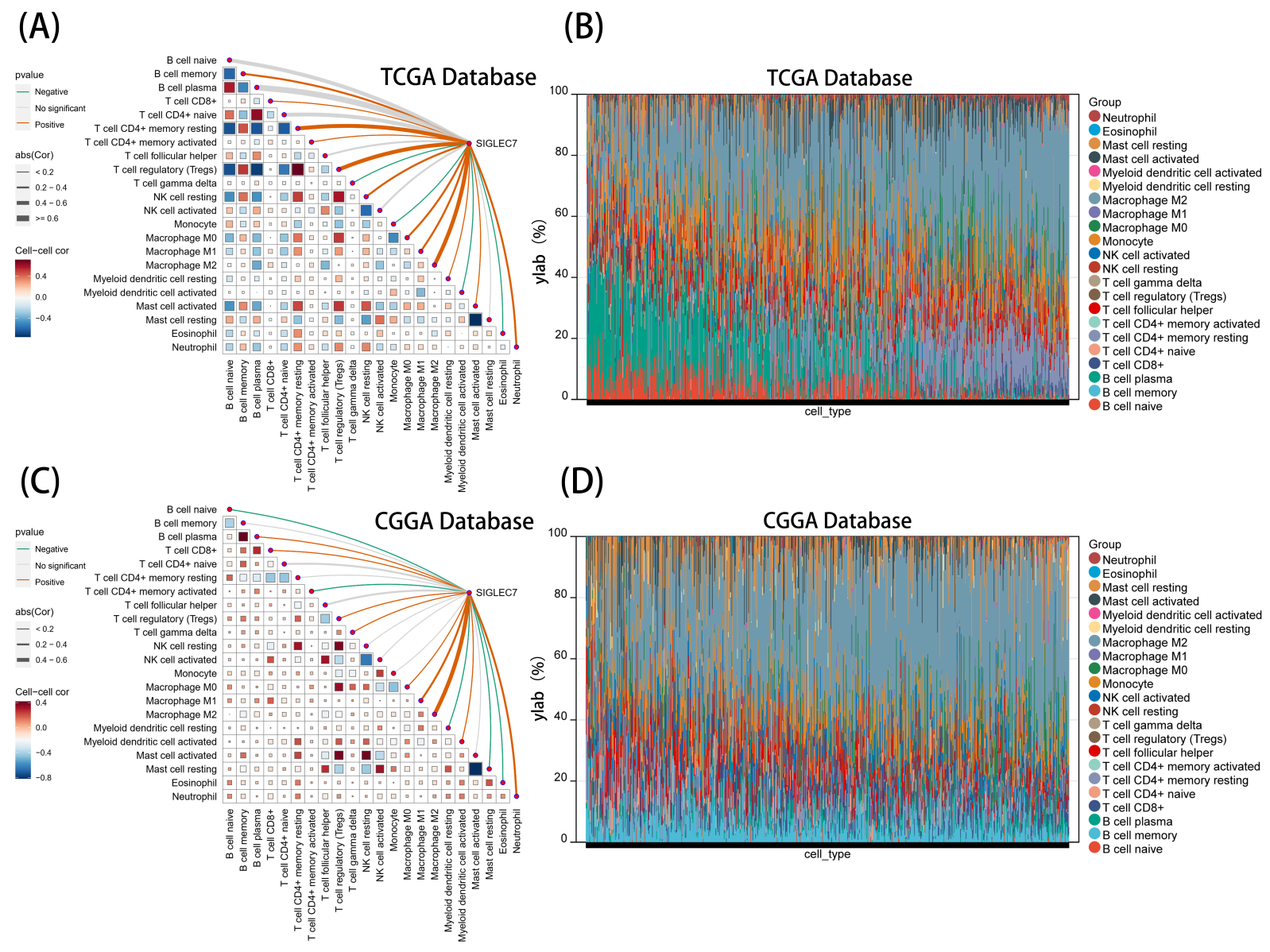


**Supplementary Figure 3**. Relationship between SIGLEC7 and immune cell infiltration. A and C, Relationship between immune cells and SIGLEC7 as well as between immune cells in TCGA and CGGA databases. B and D, Changes in the proportion of various infiltrating immune cells with the variation of SIGELCE7. From left to right, SIGLEC7 expression gradually increases.


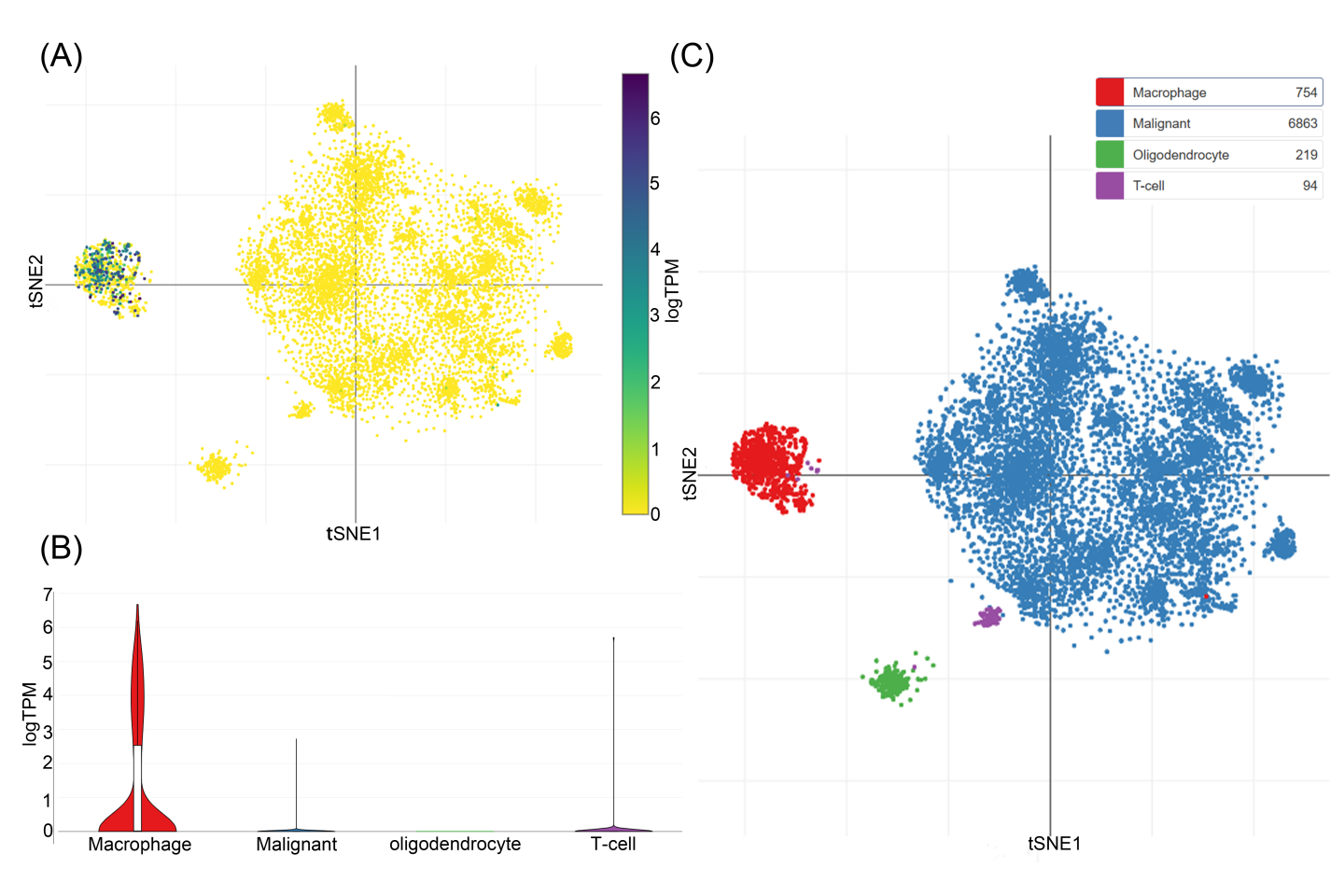


**Supplementary Figure 4**. Distribution of SIGLEC7 in the Single Cell Portal database (GSE131928). A and B, Distribution of SIGLEC7. C, Definition of cell clustering.


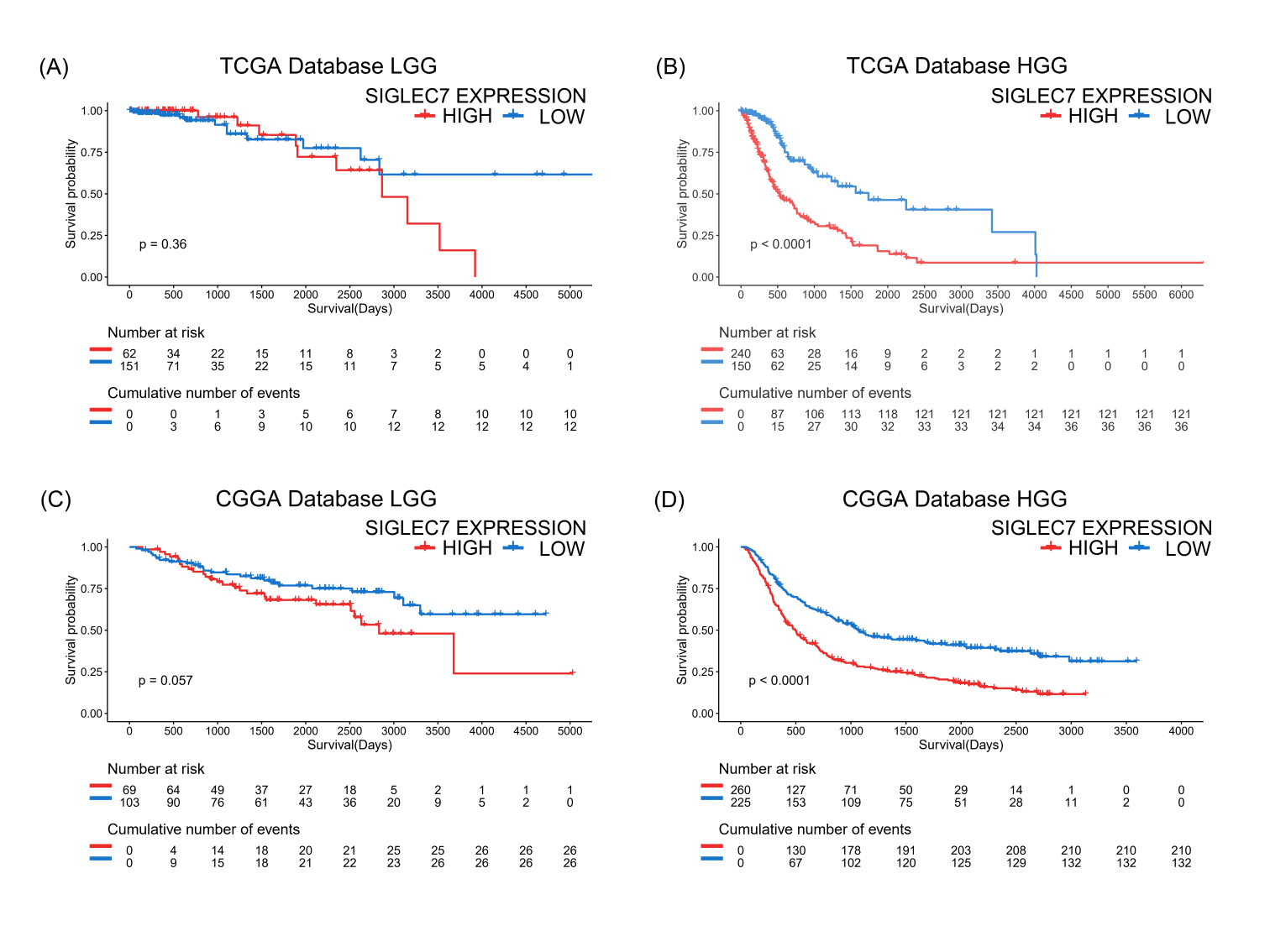


**Supplementary Figure 5**. Survival curves of high and low-grade gliomas based on SIGLEC7 expression. A and B, Prognostic analysis of SIGLEC7 high and low expression groups in different grades of gliomas in TCGA database. C and D, Prognostic analysis of SIGLEC7 high and low expression groups in different grades of gliomas in CGGA database.
