# Supplementary material for: High expression of SIGLEC7 may promote M2-type macrophage polarization leading to adverse prognosis in glioma patients
Source: Front Immunol. 2024 Aug 15;15:1411072. doi: 10.3389/fimmu.2024.1411072 (PMC11357930; doi:10.3389/fimmu.2024.1411072)
Supplement: Supplementary file 2 [file DataSheet2.docx]

Author Communication Supplementary File

Shouwei Li and Yihua An are the supervising teachers for this research, affiliated with the same institution, and have provided substantial support in terms of research resources and funding. Wenhao An, Changyuan Ren, and Zhiqiang Qiu were originally fellow disciples within the same institution, with Qiu currently pursuing studies in the United States. Lei Yuan is a former classmate of Wenhao An and has been actively engaged in academic exchanges. Many aspects of this experiment were conducted at Yihua Biotechnology Co., Ltd. in Beijing. Among them, Peishen Wang, Yanwen Cheng, Zi He, and Xinye Han are affiliated with the company (as described in the submitted manuscript) and provided significant assistance during the experimental process.


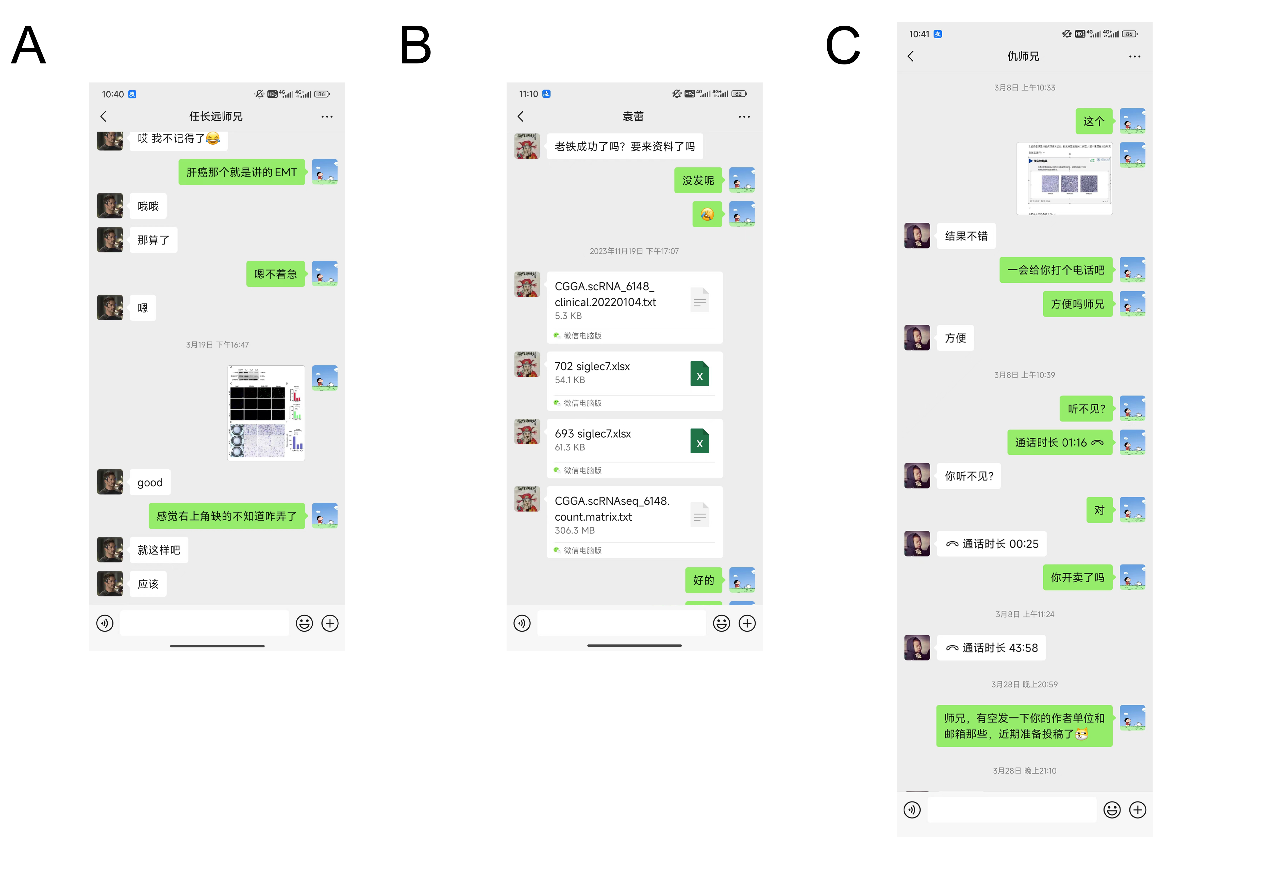


A: An exchange between Wenhao An and Changyuan Ren regarding the composition method in the manuscript, dated March 19, 2024, Beijing time. Images from the WeChat conversation can be found in the submitted manuscript.

B: An exchange between Wenhao An and Lei Yuan regarding the organization of relevant data in the article, dated November 19, 2023, Beijing time. The WeChat conversation file contains the mention of the term "SIGLEC7."

C: An exchange between Wenhao An and Zhiqiang Qiu regarding the conceptualization of the article, including several segments of voice calls. Dated March 8, 2024, Beijing time. Images from the discussion can be found correlating with the pathological figures in the submitted manuscript.


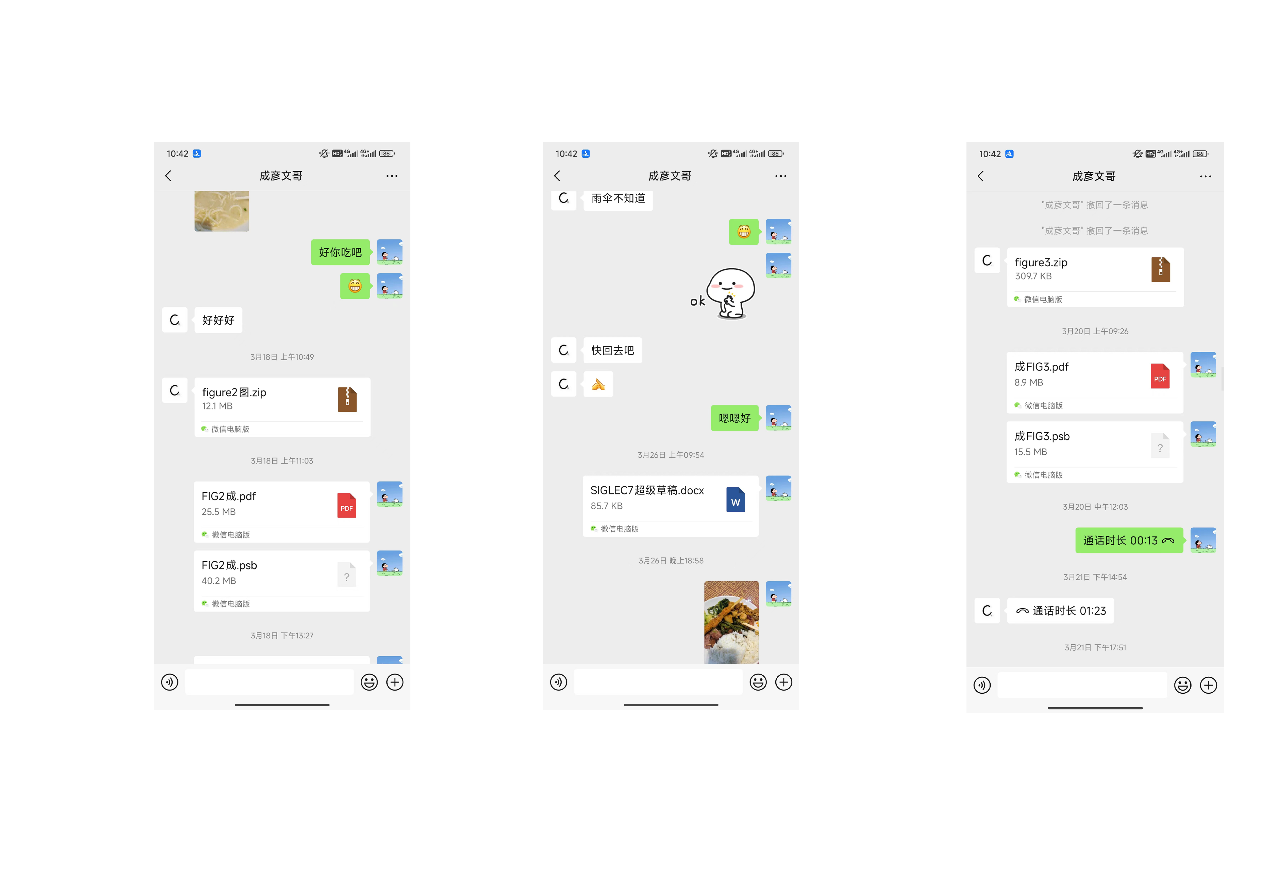


The exchange between Wenhao An and Peishen Wang, Yanwen Cheng, Zi He, and Xinye Han took place while conducting experiments at Yihua Biotechnology Co., Ltd. in Beijing. Much of the communication occurred face-to-face during the process. The presentation includes screenshots of WeChat conversations between Wenhao An and Yanwen Cheng, dated March 18th, March 26th, and March 20th respectively, Beijing time.
